# Arctic charr brain transcriptome strongly affected by summer seasonal growth but only subtly by feed deprivation

**DOI:** 10.1186/s12864-019-5874-z

**Published:** 2019-06-27

**Authors:** Anja Striberny, Even H. Jørgensen, Christophe Klopp, Elodie Magnanou

**Affiliations:** 1 Department of Arctic and Marine Biology, UiT – The Arctic University of Norway, Tromsø, Norway; 2 Plateforme Bioinformatique Toulouse, Midi-Pyrénées UBIA, INRA, Auzeville Castanet-Tolosan, France; 3 Sorbonne Université, CNRS, Biologie Intégrative des Organismes Marins, BIOM, F-66650 Banyuls-sur-Mer, France

**Keywords:** Feed deprivation, *Salvelinus alpinus*, RNA-seq, Brain transcriptome, Neuropeptides, Season

## Abstract

**Background:**

The Arctic charr (*Salvelinus alpinus*) has a highly seasonal feeding cycle that comprises long periods of voluntary fasting and a short but intense feeding period during summer. Therefore, the charr represents an interesting species for studying appetite-regulating mechanisms in fish.

**Results:**

In this study, we compared the brain transcriptomes of fed and feed deprived charr over a 4 weeks trial during their summer feeding season. Despite prominent differences in body condition between fed and feed deprived charr at the end of the trial, feed deprivation affected the brain transcriptome only slightly. In contrast, the transcriptome differed markedly over time in both fed and feed deprived charr, indicating strong shifts in basic cell metabolic processes possibly due to season, growth, temperature, or combinations thereof. The GO enrichment analysis revealed that many biological processes appeared to change in the same direction in both fed and feed deprived fish. In the feed deprived charr processes linked to oxygen transport and apoptosis were down- and up-regulated, respectively. Known genes encoding for appetite regulators did not respond to feed deprivation. Gene expression of *Deiodinase* 2 (*DIO2*), an enzyme implicated in the regulation of seasonal processes in mammals, was lower in response to season and feed deprivation. We further found a higher expression of *VGF* (non-acronymic) in the feed deprived than in the fed fish. This gene encodes for a neuropeptide associated with the control of energy metabolism in mammals, and has not been studied in relation to regulation of appetite and energy homeostasis in fish.

**Conclusions:**

In the Arctic charr, external and endogenous seasonal factors for example the increase in temperature and their circannual growth cycle, respectively, evoke much stronger responses in the brain than 4 weeks feed deprivation. The absence of a central hunger response in feed deprived charr give support for a strong resilience to the lack of food in this high Arctic species. DIO2 and VGF may play a role in the regulation of energy homeostasis and need to be further studied in seasonal fish.

**Electronic supplementary material:**

The online version of this article (10.1186/s12864-019-5874-z) contains supplementary material, which is available to authorized users.

## Background

Feeding is pivotal for animals in order to sustain their energy and substrate needs to live, grow and reproduce. In mammals, energy intake and expenditure are tightly regulated by a crosstalk of peripheral and central signalling actors and pathways [1]. Peripherally derived hunger (orexigenic) and satiety (anorexigenic) signals as well as long-term signals reporting energy status are perceived and processed in a number of brain nuclei in order to control short-term (meal-to-meal) appetite and long-term energy homeostasis [2, 3]. Of these, the arcuate nucleus (ARC) in the hypothalamus represents the pivot for controlling food intake and energy balance [4]. The ARC contains two populations of “first order” neurons, one expressing the anorexigenic proopiomelanocortin (POMC) and cocaine-and amphetamine regulated transcript (CART), the other the orexigenic agouti-related peptide (AgRP) and neuropeptide Y (NPY) [3]. These project to “second order” neurons that transduce orexigenic and anorexigenic signals via NPY and melanocortin receptors (MCR) [1]. While NPY signalling through its receptors causes an orexigenic response, signalling through MCRs results in either an anorexigenic or an orexigenic response. POMC-derived α-melanocyte-stimulating hormone (α-MSH) is a melanocortin 4 receptor (MC4R) agonist and a potent appetite suppressor in mammals [5]. AgRP, on the other hand, is an inverse agonist to the constitutively active MCRs and increases food intake [6]. These appetite and energy signalling neuropeptides have been shown to be evolutionary conserved [7, 8] and to be involved in the control of food intake in fish [9, 10]. However, responses of central appetite regulators to energy perturbation vary across species and even within species depending on the experimental design [11]. For example, *NPY* expression is higher after 7 days of feed deprivation in the hypothalamus of zebrafish (*Danio rerio*) [12] and in the preoptic area of chinook salmon (*Oncorhynchus tshawytscha*) and coho salmon (*Oncorhynchus kisutch)* [13], whereas in cunner (*Tautogolabrus adsperus*), hypothalamic *NPY* expression remains unaffected after 7 days feed deprivation [14]. Such differences in the response to feed deprivation are not unexpected as fish represent the most diverse group of vertebrates with a myriad of adaptations to spatially different and temporal changing environments. For example, in the high-latitude inhabiting anadromous (sea-migrating) Arctic charr (*Salvelinus alpinus*), food intake varies dramatically from little or no feeding while residing in fresh water during winter to voracious feeding during their short summer residence in the sea [15, 16]. This behaviour, which presumably developed as a response to seasonal and spatial differences in water temperature and food availability at high latitudes, now appears to be regulated independently of these factors; captive offspring of anadromous Arctic charr exhibit pronounced seasonal changes in appetite and growth when held at constant temperature and given food in excess [17]. Despite such seasonal changes in food intake, the expressions of orexigenic and anorexigenic appetite regulators in different brain regions is similar between anorexic winter charr with hyperphagic summer charr [18]. Furthermore, the expressions of orexigenic and anorexigenic neuropeptides in the hypothalamus are unaffected by short- and long-term feed deprivation in this species [19]. However, in these and in most other studies investigating appetite regulation in fish, expression levels of known appetite regulators have been measured by RT-qPCR, a method with the drawback of restricting the focus to a limited number of genes. The fact that novel actors in the complex control of food intake are still being discovered calls for a more global approach when investigating appetite regulation in fish. Today, high-throughput RNA sequencing is a powerful tool in experimental biology. Transcriptomic approaches have been applied in various contexts to improve knowledge of the biology of the seasonal Arctic charr [20, 21], albeit not with focus on appetite regulation. Consequently, we sequenced the brain transcriptome of fed and feed deprived charr during their natural summertime growth in an attempt to advance the knowledge on global responses to feed deprivation, assess alterations of known central appetite regulators, and to identify possible new actors involved in the control of appetite and energy metabolism in fish.

## Results

### Morphometrics

**Fig. 1:**
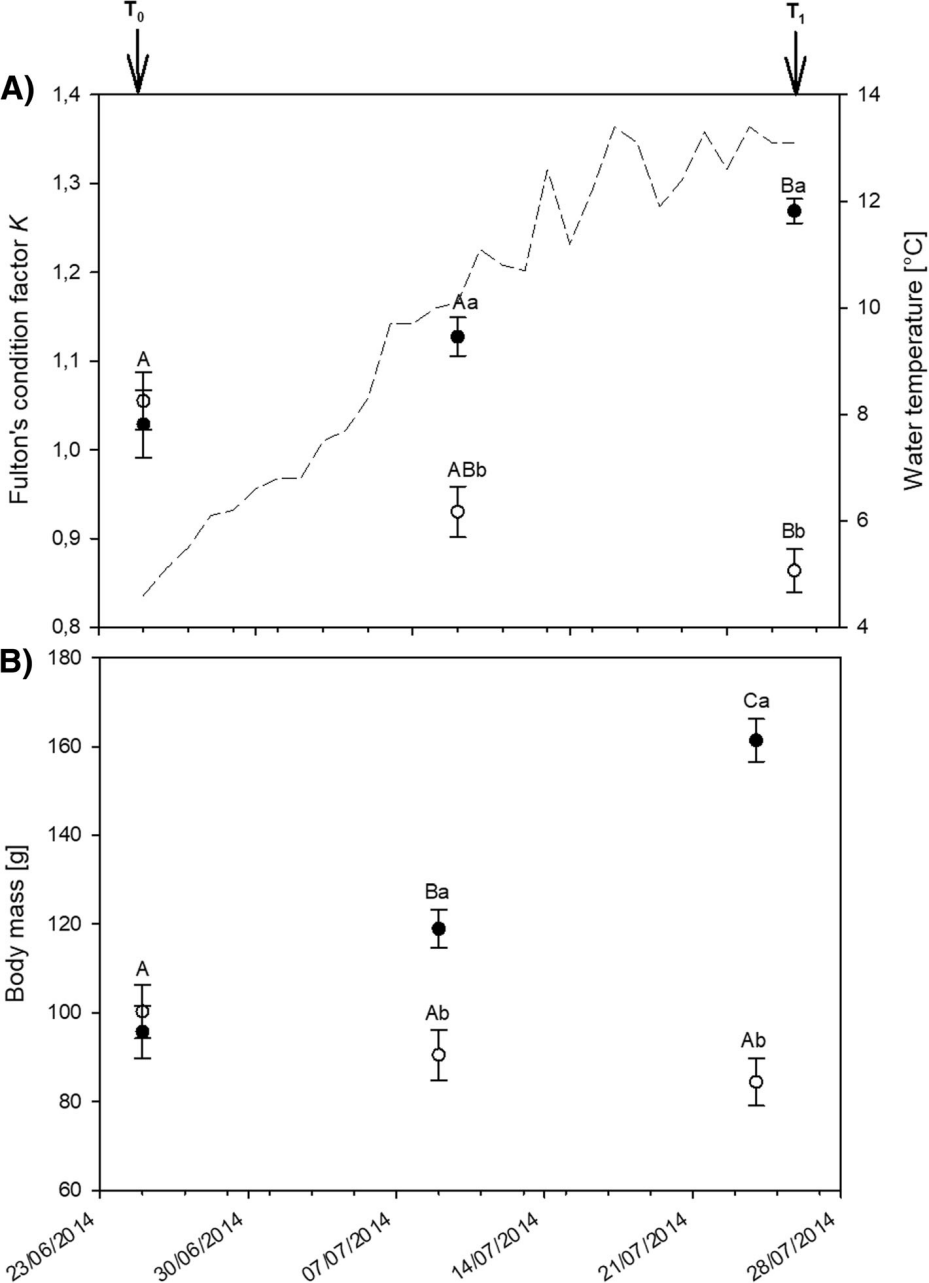
Fulton’s condition factor (**a**) and body mass (**b**) of tattooed fed (black dots) and feed deprived Arctic charr (white dots) during the experiment. Charr sampled at T_0_ were measured before distribution to Tank 1 and Tank 2. Dashed line: water temperature. *n* = 21 per treatment group. Values are shown as mean ± SEM. Different capital and lowercase letters denote differences within treatment group at different time points and differences between treatment groups at the given time point, respectively

The experiment was performed on two-year old immature anadromous Arctic charr held under natural photoperiod (24 h light at that time of the year) and ambient water temperature. Growth development during the 4 weeks experimental period was assessed on subsamples of 15 tagged fish per treatment group. Average body mass and condition factor were 98.0 ± 4.2 g and 1.04 ± 0.02 on June 25, 2014, the start of the experiment (T_0_) (Fig. 1). On July 23, the end of the experiment (T_1_), body mass and condition factor were markedly higher in fed (Fed) than in feed deprived (FDP) charr with 161.4 ± 4.7 g and 1.27 ± 0.01, and 84.4 ± 5.2 g and 0.86 ± 0.02 in the fed and FDP charr, respectively. Average body mass and condition factor of the 5 fish sampled for transcriptomics fell in the same range as for the tagged fish and were 108.3 ± 10.2 g and 1.05 ± 0.03 at T_0_ and 157.2 ± 8.3 g and 1.25 ± 0.03 (Fed) and 80.6 ± 6.3 g and 0.92 ± 0.04 (FDP) at T_1_.

### De novo transcriptome assembly and annotation

The de novo brain transcriptome assembly produced 49829 contigs with a FPKM greater than 1 for at least one of the 15 samples. Their total length equalled 84028148 base pairs. The N50 (i.e., the contig length that produces half the bases of the assembly) reached 2663 bp (Table 1).

**Table 1:** General statistics of contigs generated by RNAseq technology for brain gene expression characterization. Only contigs possessing a FPKM greater than 1 for at least one library were considered for annotation and further expression analysis

Number of base pairs in reads	42491174498
Number of reads	420704698
Number of base pairs in contigs (FPKM> 1)	84028148
Number of contigs (FPKM> 1)	49829
N50	2663
N90	816
Number of putative micro-satellites	34440
Number of putative SNPs	420406
Number of contigs including SNP	39484

**Fig. 2:**
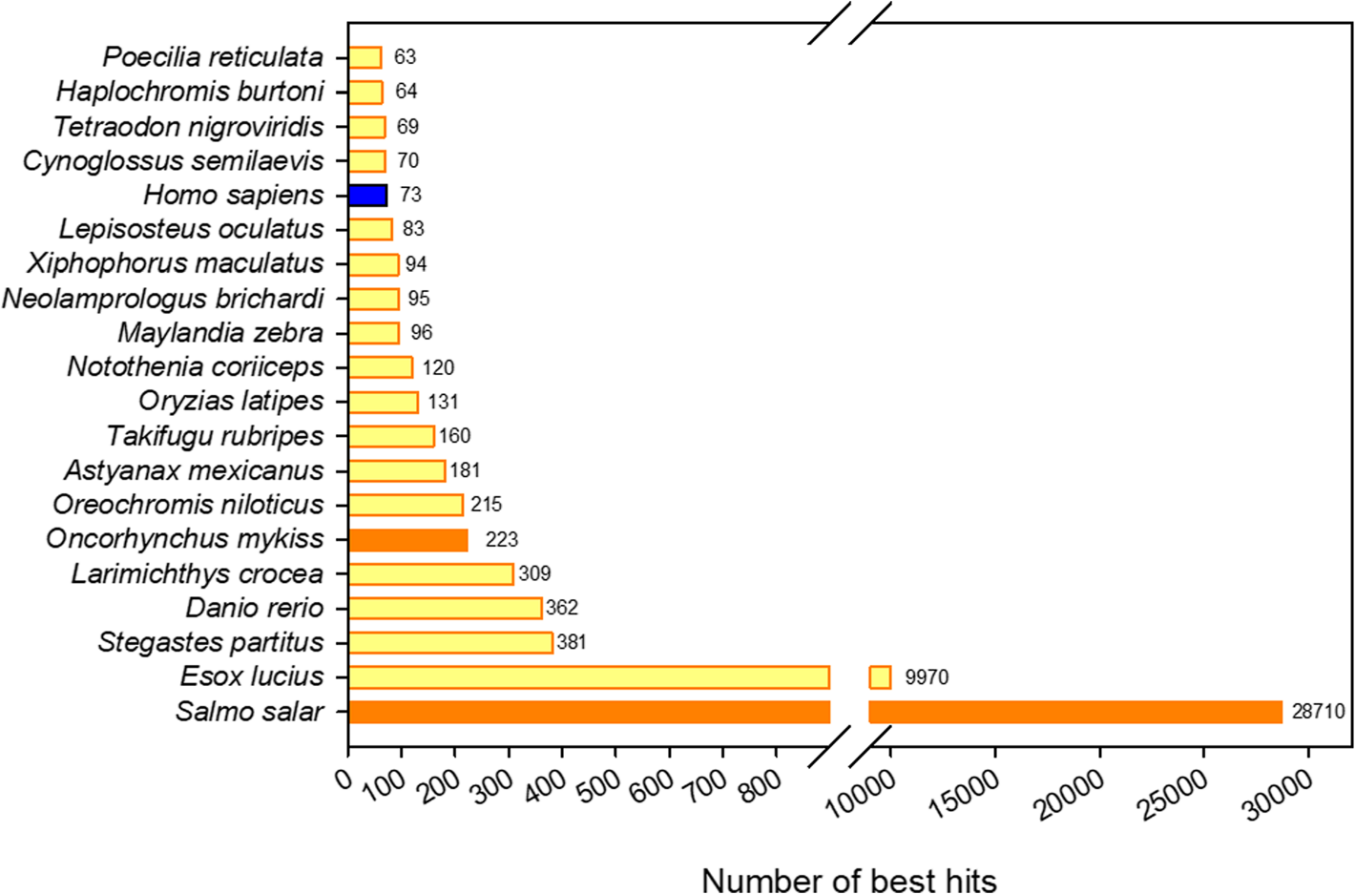
Top 20 species contributing the most to best-hit annotations. Best hits were based on all databases involved in the annotation process. Yellow: teleosts, orange: salmonids, blue: mammals

The annotation rate reached 85.07% of the 49829 contigs. The Atlantic salmon (*Salmo salar*) contributed most to the annotation of the Arctic charr brain transcriptome with 57.6% of the contigs annotated on this species (Fig. 2). All other species contributing to the annotation, except *Homo sapiens,* were exclusively teleosts including another salmonid, the rainbow trout (*Oncorhynchus mykiss*). One or more GO identifier could be assigned to 13231 out of the 49829 contigs. Different approaches were used to verify the quality of the contigs. First, the assembly quality and annotation completeness of the transcriptome were assessed by BUSCO analysis. Out of 4584 single-copy ortholog genes common to Actinopterygii, the assembly was 67.2% complete (2560 complete single-copy BUSCOs and 524 complete duplicated BUSCOs), while only 2.8% of contigs were fragmented (130 BUSCOs) and 30.0% were missing (1370 BUSCOs). Second, a comparison to a phylogenetically close species reference proteome was achieved using the Atlantic salmon database from the NCBI. Out of 97555 *Salmo salar* proteins, 47419 were aligned with at least 80% identity over 80% of their length on our assembly, which corresponded to 12238 Arctic charr contigs. Third, initial reads were mapped to the contigs in order to validate to what extent the contigs reflected the initial information. The contigs had high realignment rates that ranged between 88 and 89% depending on the sample (15 libraries). Furthermore, the construction of the RNAseq data set was verified by mapping the reads from an Arctic charr gill transcriptome [22] on our set of contigs. On average, 79.35% of reads from this gill transcriptome (SRA accession: SRX314607) were aligned to the 15 Arctic charr libraries. Finally, both proteins and RNAs from the *S. alpinus* reference genome (NCBI ID: 12179) [23] were aligned to our set of contigs. Out of 59926 proteins, 49212 (82%) had a hit on the contigs, 31316 of these proteins shared over 80% similarity and 80% of coverage with the built contigs. Regarding RNAs from the reference genome, 76% (51217 out of the 67196) had a hit on our contigs, with 24432 RNAs having over 80% of similarity and 80% of coverage with the contigs.

### Gene expression patterns and differential gene expression

**Fig. 3:**
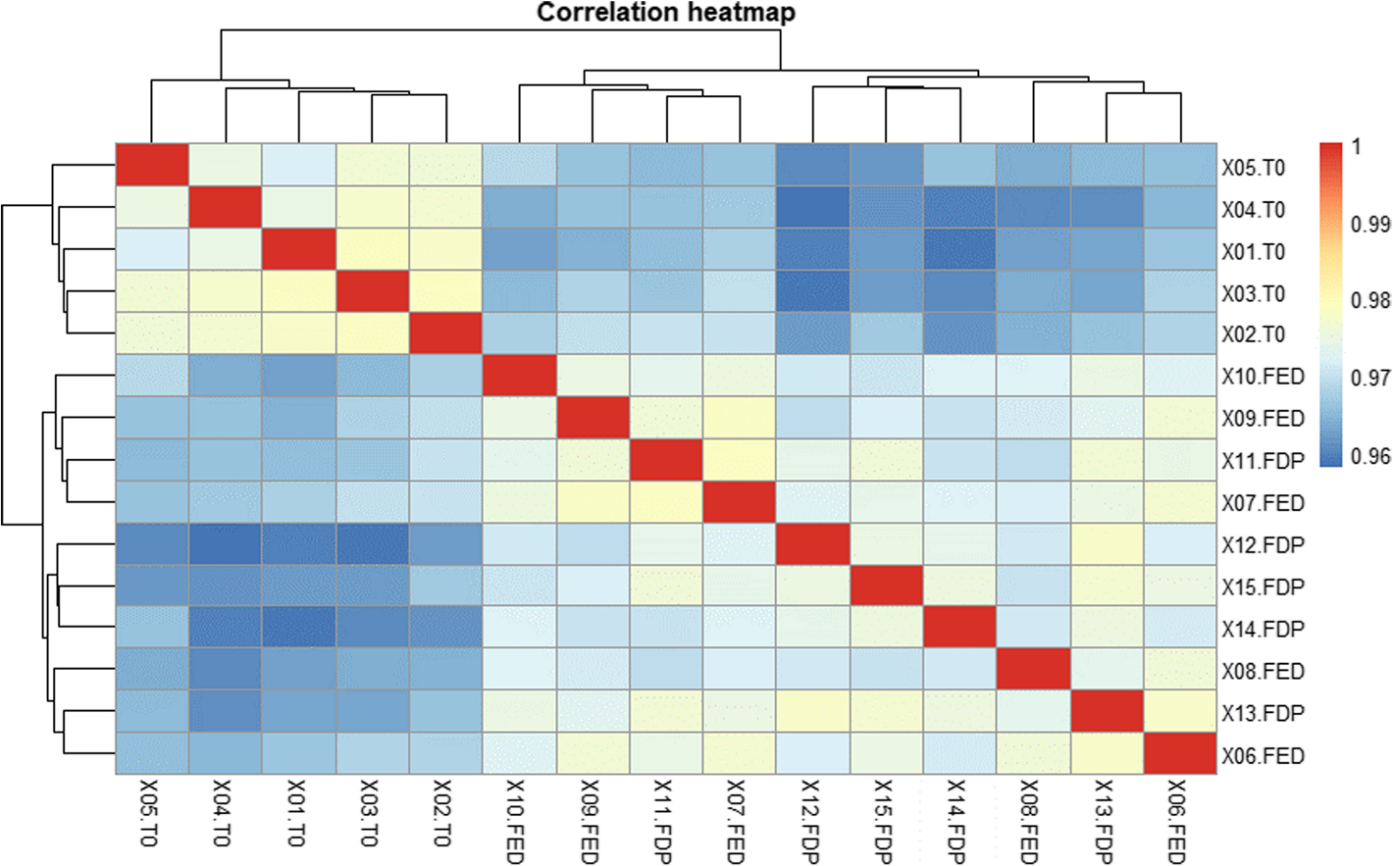
Correlation heatmap based on raw counts of the 49829 contigs possessing a FPKM greater than 1 for at least one sample. X01-X15: number of sampled fish during the experiment. T0: fish sampled at the start of the experiment (June 25). FED: fed fish sampled at the end of the experiment (July 23). FDP: feed deprived fish sampled at the end of experiment (July 23)

In order to inspect the overall expression patterns of the 15 samples, a correlation heatmap based on the raw counts of the 49829 contigs possessing a FPKM greater than 1 for at least one sample was drawn (Fig. 3). This analysis highlighted that all samples from T_0_ clustered together and were markedly different from samples taken at T_1_. Samples of the fed and feed deprived charr at T_1_ did not cluster in accordance with the treatment group (Fig. 3).

In fed Arctic charr, 2819 contigs differed over time (Table 2). This corresponded to 5.7% of all sequenced contigs. Among these, 1534 contigs were up-regulated, while 1285 were down-regulated.

**Table 2:** Number of up- and down-regulated transcripts in the different comparisons returned by EdgeR analysis. Cut-off at FDR < 0.05 and at LogFC 0.5/−0.5

T_1__Fed versus T_0_		T_1__FDP versus T_0_		T_1__ FDP versus T_1__Fed	
Up	Down	Up	Down	Up	Down
1534	1285	2616	1954	68	107

**Fig. 4:**
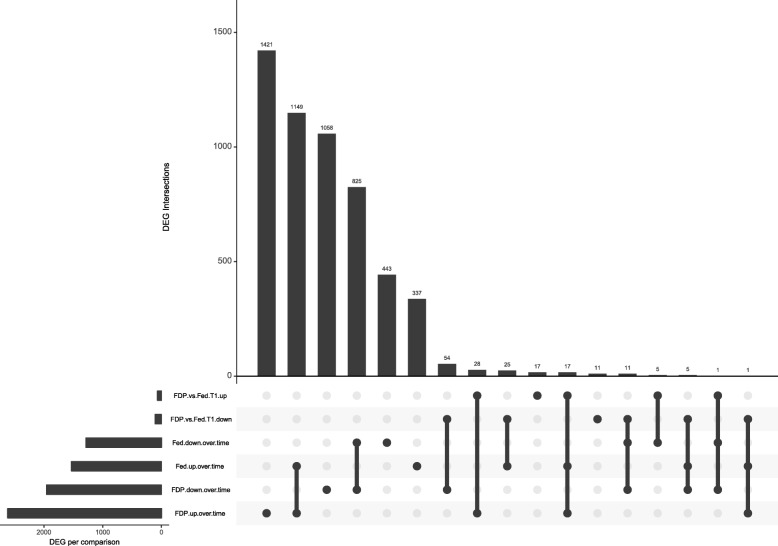
Number of up- and down-regulated transcripts in the different pairwise comparisons. The total number of differentially expressed transcripts for the different comparisons are presented as horizontal bars. Vertical bars represent lists of differentially expressed genes that were only found in one specific comparison or shared by two or more comparisons, indicated by dots and dots connected by lines, respectively, below

In feed deprived charr, 4570 (9.2%) contigs differed over time (Table 2). Of these 2616 were up-regulated while 1954 were down-regulated from start to the end of experiment. At T_1_, only 175 contigs (0.4% of all contigs) were found to be differentially expressed between FDP and Fed charr (Table 2), with 68 contigs being up-regulated and 107 being down-regulated. Matching the six lists of up- and down regulated contigs from the three comparisons in Up-set graphs enabled us to break down the lists of differentially expressed genes and find the intersection points of all three comparisons (Fig. 4). Over time, 1449 contigs were up-regulated and 825 down-regulated in both FDP and fed charr. Interestingly, at the same time, 1421 contigs were uniquely up-regulated in the FDP versus T_0_ comparison.

In contrast, only 825 contigs were uniquely up-regulated in fed charr over time. Similarly, 1058 contigs, were uniquely down-regulated in FDP charr over time, whereas only 337 contigs were uniquely down-regulated contigs in the Fed versus T_0_ comparison. Chi-square tests of the 2 × 2 contingency tables of up- and downregulated genes indicated that there was a correlation of the proportion between uniquely and shared differentially expressed contigs and feeding regime over time (up-regulated: X-squared = 387.74, *p*-value < 0.0001, downregulated: X-squared = 153.5, *p*-value < 0.0001) (Fig. 4). Only five contigs were up-regulated in the Fed vs T_0_ comparison whilst down regulated in the FDP versus T_0_ comparison. No contigs were down-regulated in the Fed vs T_0_ and at the same time up-regulated in the FDP vs T_0_ comparison (Fig. 4).

GO enrichment analysis for biological processes (BP) was employed to study the biological functions of the differentially expressed contigs. This would depict global effects of season and feed deprivation during summer on the charr’s brain transcriptome.

Contigs that were only up-regulated in fed fish over time were associated with biological processes that included oxygen transport and protein related biological processes (Table 3). Down-regulated contigs in fed fish contributed amongst others to terms like ion transport, protein complex assembly (Table 4), and “feeding behaviour” (Additional file 4: Table S2). In both fed and feed deprived charr, we found that up-regulated contigs over time were involved in basic cell metabolism processes such as DNA replication and RNA metabolism (Table 5). Contigs that were down-regulated over time regardless of the feeding regime were amongst others related to ion transport, protein related processes and wnt signaling (Table 6, Additional file 6: Table S4). Feed deprivation over time led to an up-regulation of contigs relating to processes such as catabolism, apoptosis and immune system (Table 7) and a down-regulation of e.g. oxygen transport (Table 8). An up-regulation of contigs that take part in catabolic and apoptotic processes and down-regulation of oxygen transport was also found in the endpoint comparison between FDP and fed charr (Additional file 9: Table S7, Table 9, Additional file 10: Table S8).

**Table 3:** Biological processes enriched by up-regulated contigs only found in Fed versus T_0_ (see yellow fraction in Venn diagram Additional file 1: Fig. S1). Terms sorted by the number of contributing contigs

GO.ID	Term	Annotated	Significant	Expected	*p*-value
GO:0006810	Transport	1483	25	14.54	0.0026
GO:0051234	establishment of localization	1484	25	14.55	0.0026
GO:0051179	Localization	1501	25	14.71	0.0031
GO:0044765	single-organism transport	883	19	8.66	0.0006
GO:1902578	single-organism localization	893	19	8.75	0.0007
GO:0015669	gas transport	16	10	0.16	3.20E-17
GO:0015671	oxygen transport	16	10	0.16	3.20E-17
GO:0006457	protein folding	104	5	1.02	0.0034

**Table 4:** Biological processes enriched by down-regulated contigs only found in Fed versus T_0_ (see yellow fraction in Venn diagram Additional file 1: Figure S1) Terms sorted by the number of contributing contigs

GO.ID	Term	Annotated	Significant	Expected	*p*-value
GO:0044765	single-organism transport	883	11	5.77	0.02405
GO:1902578	single-organism localization	893	11	5.84	0.02593
GO:0006811	ion transport	532	10	3.48	0.0019
GO:0006461	protein complex assembly	173	4	1.13	0.0258
GO:0070271	protein complex biogenesis	173	4	1.13	0.0258
GO:0065003	macromolecular complex assembly	189	4	1.24	0.03419
GO:0071822	protein complex subunit organization	189	4	1.24	0.03419
GO:0009966	regulation of signal transduction	212	4	1.39	0.04877
GO:0010646	regulation of cell communication	213	4	1.39	0.04947
GO:0023051	regulation of signalling	213	4	1.39	0.04947

**Table 5:** Biological processes enriched by up-regulated contigs found in Fed versus T_0_ and FDP versus T_0_ (see white fraction in Venn diagram Additional file 1: Figure S1). Terms sorted by the number of contributing contigs

GO.ID	Term	Annotated	Significant	Expected	*p*-value
GO:0019438	aromatic compound biosynthetic process	782	28	16	0.0021
GO:0018130	heterocycle biosynthetic process	796	28	16.28	0.0027
GO:1901362	organic cyclic compound biosynthetic process	803	28	16.43	0.0031
GO:0034654	nucleobase-containing compound biosynthetic process	750	25	15.34	0.0092
GO:0080090	regulation of primary metabolic process	622	24	12.72	0.0017
GO:0031323	regulation of cellular metabolic process	632	24	12.93	0.0021
GO:0019222	regulation of metabolic process	641	24	13.11	0.0025
GO:0019219	regulation of nucleobase-containing compound metabolic process	577	23	11.8	0.0014
GO:0051171	regulation of nitrogen compound metabolic process	587	23	12.01	0.0017
GO:0060255	regulation of macromolecule metabolic process	619	23	12.66	0.0034
GO:0006355	regulation of transcription, DNA-templated	568	22	11.62	0.0025
GO:1903506	regulation of nucleic acid-templated transcription	568	22	11.62	0.0025
GO:2001141	regulation of RNA biosynthetic process	569	22	11.64	0.0026
GO:0051252	regulation of RNA metabolic process	572	22	11.7	0.0028
GO:0010556	regulation of macromolecule biosynthetic process	580	22	11.87	0.0033
GO:2000112	regulation of cellular macromolecule biosynthetic process	580	22	11.87	0.0033
GO:0031326	regulation of cellular biosynthetic process	581	22	11.89	0.0034
GO:0009889	regulation of biosynthetic process	582	22	11.91	0.0034
GO:0010468	regulation of gene expression	583	22	11.93	0.0035
GO:0006351	transcription, DNA-templated	630	22	12.89	0.0087
GO:0097659	nucleic acid-templated transcription	630	22	12.89	0.0087
GO:0032774	RNA biosynthetic process	633	22	12.95	0.0092
GO:0051276	chromosome organization	94	7	1.92	0.003
GO:0006325	chromatin organization	72	6	1.47	0.0034
GO:0006955	immune response	50	5	1.02	0.0034
GO:0002376	immune system process	52	5	1.06	0.004
GO:0016571	histone methylation	11	3	0.23	0.0012
GO:0018022	peptidyl-lysine methylation	11	3	0.23	0.0012
GO:0034968	histone lysine methylation	11	3	0.23	0.0012
GO:0018205	peptidyl-lysine modification	22	3	0.45	0.0097
GO:0016569	covalent chromatin modification	23	3	0.47	0.011
GO:0016570	histone modification	23	3	0.47	0.011
GO:0019882	antigen processing and presentation	26	3	0.53	0.0154
GO:0006479	protein methylation	27	3	0.55	0.0171
GO:0008213	protein alkylation	27	3	0.55	0.0171
GO:0033993	response to lipid	28	3	0.57	0.0189
GO:0043401	steroid hormone mediated signaling pathway	28	3	0.57	0.0189
GO:0048545	response to steroid hormone	28	3	0.57	0.0189
GO:0071383	cellular response to steroid hormone stimulus	28	3	0.57	0.0189
GO:0071396	cellular response to lipid	28	3	0.57	0.0189
GO:0014070	response to organic cyclic compound	29	3	0.59	0.0208
GO:0071407	cellular response to organic cyclic compound	29	3	0.59	0.0208
GO:0009725	response to hormone	31	3	0.63	0.0248
GO:0009755	hormone-mediated signaling pathway	31	3	0.63	0.0248
GO:0032870	cellular response to hormone stimulus	31	3	0.63	0.0248
GO:0018193	peptidyl-amino acid modification	34	3	0.7	0.0316
GO:0006334	nucleosome assembly	35	3	0.72	0.0341
GO:0016568	chromatin modification	35	3	0.72	0.0341
GO:0031497	chromatin assembly	35	3	0.72	0.0341
GO:0034728	nucleosome organization	35	3	0.72	0.0341
GO:0006323	DNA packaging	36	3	0.74	0.0366
GO:0006333	chromatin assembly or disassembly	37	3	0.76	0.0393

**Table 6:** Biological processes enriched by down-regulated contigs found in Fed versus T_0_ and FDP versus T_0_ (see white fraction in Venn diagram Additional file 1: Figure S1). Terms sorted by the number of contributing contigs

GO.ID	Term	Annotated	Significant	Expected	*p*-value
GO:0044699	single-organism process	3629	54	45.37	0.04
GO:0044765	single-organism transport	883	17	11.04	0.0443
GO:1902578	single-organism localization	893	17	11.16	0.0485
GO:0006811	ion transport	532	12	6.65	0.0322
GO:0006470	protein dephosphorylation	126	6	1.58	0.0048
GO:0016311	dephosphorylation	162	6	2.03	0.0156
GO:0015672	monovalent inorganic cation transport	175	6	2.19	0.0219
GO:0006813	potassium ion transport	88	4	1.1	0.024
GO:0006457	protein folding	104	4	1.3	0.0408
GO:0051258	protein polymerization	36	3	0.45	0.01

**Table 7:** Biological processes enriched by up-regulated contigs only found in FDP versus T_0_ (see blue fraction in Venn diagram Additional file 1: Figure S1). Terms sorted by the number of contributing contigs

GO.ID	Term	Annotated	Significant	Expected	*p*-value
GO:0065007	biological regulation	2021	62	47.66	0.00925
GO:0050789	regulation of biological process	1973	61	46.53	0.00837
GO:0050794	regulation of cellular process	1934	59	45.61	0.01308
GO:0006725	cellular aromatic compound metabolic process	1375	42	32.43	0.03914
GO:0090304	nucleic acid metabolic process	1097	35	25.87	0.03448
GO:0016070	RNA metabolic process	884	30	20.85	0.02407
GO:0060255	regulation of macromolecule metabolic process	619	22	14.6	0.03315
GO:0080090	regulation of primary metabolic process	622	22	14.67	0.03473
GO:0031323	regulation of cellular metabolic process	632	22	14.9	0.04041
GO:0019222	regulation of metabolic process	641	22	15.12	0.04609
GO:0051252	regulation of RNA metabolic process	572	20	13.49	0.04778
GO:0006396	RNA processing	189	10	4.46	0.01367
GO:0006955	immune response	50	7	1.18	0.00015
GO:0002376	immune system process	52	7	1.23	0.0002
GO:0008219	cell death	58	6	1.37	0.00231
GO:0016265	death	58	6	1.37	0.00231
GO:0010941	regulation of cell death	45	5	1.06	0.00391
GO:0042981	regulation of apoptotic process	45	5	1.06	0.00391
GO:0043067	regulation of programmed cell death	45	5	1.06	0.00391
GO:0006915	apoptotic process	57	5	1.34	0.0107
GO:0012501	programmed cell death	57	5	1.34	0.0107
GO:0048518	positive regulation of biological process	67	5	1.58	0.02048
GO:0006397	mRNA processing	68	5	1.6	0.0217
GO:0016071	mRNA metabolic process	79	5	1.86	0.03824
GO:0019882	antigen processing and presentation	26	4	0.61	0.00297
GO:0051726	regulation of cell cycle	40	4	0.94	0.01409
GO:0015074	DNA integration	55	4	1.3	0.04013
GO:0010942	positive regulation of cell death	13	3	0.31	0.0031
GO:0043065	positive regulation of apoptotic process	13	3	0.31	0.0031
GO:0043068	positive regulation of programmed cell death	13	3	0.31	0.0031
GO:0007050	cell cycle arrest	18	3	0.42	0.0081
GO:0045786	negative regulation of cell cycle	21	3	0.5	0.01254
GO:0008380	RNA splicing	26	3	0.61	0.0225

**Table 8:** Biological processes enriched by down-regulated contigs only found FDP versus T_0_ (see blue fraction in Venn diagram Additional file 1: Figure S1). Terms sorted by the number of contributing contigs

GO.ID	Term	Annotated	Significant	Expected	*p*-value
GO:0006807	nitrogen compound metabolic process	1736	66	53.76	0.03251
GO:1901360	organic cyclic compound metabolic process	1400	55	43.36	0.0299
GO:0009058	biosynthetic process	1439	55	44.57	0.04759
GO:1901576	organic substance biosynthetic process	1374	54	42.55	0.03127
GO:0044249	cellular biosynthetic process	1358	53	42.06	0.03699
GO:0006725	cellular aromatic compound metabolic process	1375	53	42.58	0.0453
GO:1901564	organonitrogen compound metabolic process	620	28	19.2	0.02644
GO:0016043	cellular component organization	466	23	14.43	0.01725
GO:0071840	cellular component organization or biogenesis	499	23	15.45	0.03484
GO:1901566	organonitrogen compound biosynthetic process	451	21	13.97	0.039
GO:0006508	proteolysis	298	18	9.23	0.00495
GO:0022607	cellular component assembly	222	13	6.88	0.02012
GO:0034622	cellular macromolecular complex assembly	127	12	3.93	0.00054
GO:0007017	microtubule-based process	132	12	4.09	0.00076
GO:0006461	protein complex assembly	173	12	5.36	0.00728
GO:0070271	protein complex biogenesis	173	12	5.36	0.00728
GO:0065003	macromolecular complex assembly	189	12	5.85	0.01414
GO:0071822	protein complex subunit organization	189	12	5.85	0.01414
GO:0006082	organic acid metabolic process	226	12	7	0.04764
GO:0019752	carboxylic acid metabolic process	226	12	7	0.04764
GO:0043436	oxoacid metabolic process	226	12	7	0.04764
GO:0006520	cellular amino acid metabolic process	126	10	3.9	0.00554
GO:0051258	protein polymerization	36	9	1.11	1.00E-06
GO:0043623	cellular protein complex assembly	76	9	2.35	0.00052
GO:0006457	protein folding	104	9	3.22	0.00477
GO:0030163	protein catabolic process	118	8	3.65	0.02963
GO:1902582	single-organism intracellular transport	88	7	2.73	0.01889
GO:0044257	cellular protein catabolic process	100	7	3.1	0.03502
GO:0051603	proteolysis involved in cellular protein catabolic process	100	7	3.1	0.03502
GO:1901605	alpha-amino acid metabolic process	58	5	1.8	0.03302
GO:0006270	DNA replication initiation	4	4	0.12	9.00E-07
GO:0006261	DNA-dependent DNA replication	5	4	0.15	4.40E-06
GO:0006839	mitochondrial transport	16	4	0.5	0.00121
GO:0009069	serine family amino acid metabolic process	20	4	0.62	0.00293
GO:0008652	cellular amino acid biosynthetic process	31	4	0.96	0.01461
GO:1901607	alpha-amino acid biosynthetic process	31	4	0.96	0.01461
GO:0071103	DNA conformation change	44	4	1.36	0.04628
GO:0009070	serine family amino acid biosynthetic process	7	3	0.22	0.00094
GO:0031032	actomyosin structure organization	7	3	0.22	0.00094
GO:0000278	mitotic cell cycle	14	3	0.43	0.00828
GO:0006720	isoprenoid metabolic process	14	3	0.43	0.00828
GO:0008299	isoprenoid biosynthetic process	14	3	0.43	0.00828
GO:1903047	mitotic cell cycle process	14	3	0.43	0.00828
GO:0015669	gas transport	16	3	0.5	0.01218
GO:0015671	oxygen transport	16	3	0.5	0.01218

**Table 9:** Biological processes enriched by down-regulated contigs comparing feed deprived versus fed charr at end of experiment. Terms sorted by the number of contributing contigs

GO.ID	Term	Annotated	Significant	Expected	*p*-value
GO:0006810	transport	1483	15	8.85	0.02027
GO:0051234	establishment of localization	1484	15	8.85	0.02039
GO:0051179	localization	1501	15	8.96	0.0225
GO:0044765	single-organism transport	883	14	5.27	0.00038
GO:1902578	single-organism localization	893	14	5.33	0.00043
GO:0015669	gas transport	16	11	0.1	3.50E-22
GO:0015671	oxygen transport	16	11	0.1	3.50E-22
GO:0006259	DNA metabolic process	228	6	1.36	0.00214
GO:0006260	DNA replication	113	5	0.67	0.00052
GO:0006270	DNA replication initiation	4	4	0.02	1.10E-09
GO:0006261	DNA-dependent DNA replication	5	4	0.03	5.50E-09
GO:0051258	protein polymerization	36	4	0.21	5.60E-05
GO:0043623	cellular protein complex assembly	76	4	0.45	0.00103
GO:0034622	cellular macromolecular complex assembly	127	4	0.76	0.00666
GO:0006461	protein complex assembly	173	4	1.03	0.01908
GO:0070271	protein complex biogenesis	173	4	1.03	0.01908
GO:0065003	macromolecular complex assembly	189	4	1.13	0.02546
GO:0071822	protein complex subunit organization	189	4	1.13	0.02546
GO:0022607	cellular component assembly	222	4	1.32	0.04232
GO:0007017	microtubule-based process	132	3	0.79	0.04352

In an attempt to unravel whether central appetite signalling pathways in the charr brain transcriptome were modulated by feed-deprivation, we screened the lists of differentially expressed contigs systematically for candidate genes that have previously been demonstrated to be involved in the regulation of appetite and energy homeostasis in fish [9, 10]. Further, due to a strong effect of season and temperature seen on the brain transcriptome (Fig. 3), genes involved in seasonal rhythms were included in the search. Lastly, we searched the dataset for possible new actors involved in the regulation of energy homeostasis and food intake, not previously described in fish.

**Fig. 5:**
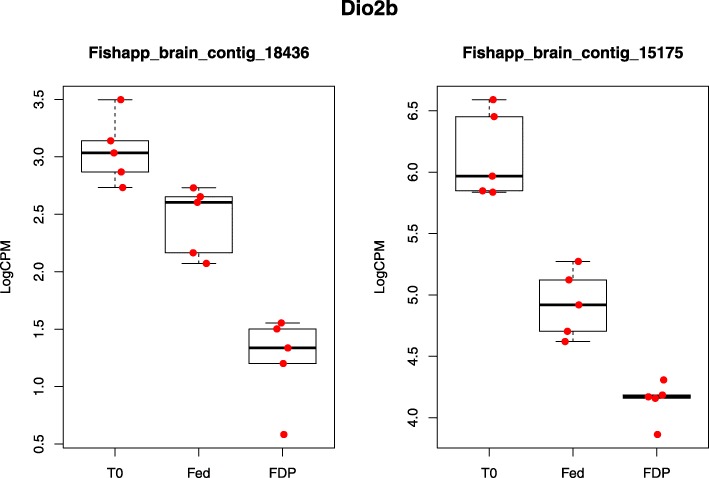
Log counts per million reads (logCPM) for contigs corresponding to Dio2b in the three treatment groups. Data are presented as box and whisker plots with median, 25th and 75th percentiles and 1.5 * interquartile range. In addition, individual data points are indicated within the plot

**Fig. 6:**
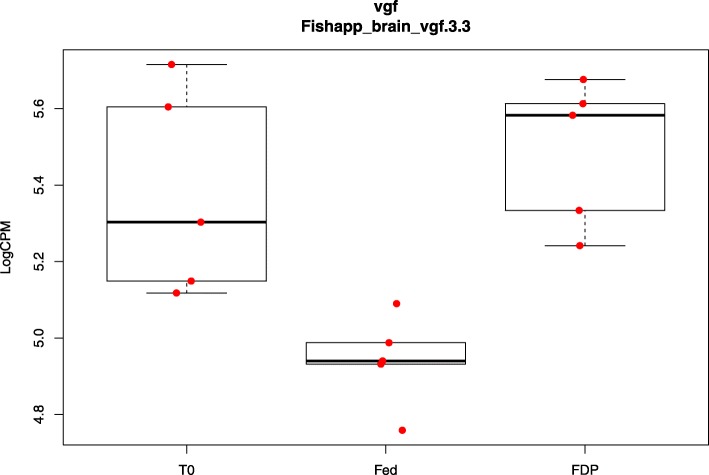
Log counts per million reads (logCPM) for the contig vgf3.3 in the three treatment groups. Data are presented as box and whisker plots with median, 25th and 75th percentiles and 1.5 * interquartile range. In addition, individual data points are indicated within the plot

Several candidate genes possibly involved in seasonality and food intake control in fish were found to be differentially expressed between one or several comparisons and are displayed in Table 10. Differences in expression were mainly found between the T_1_ and T_0_ for FDP and/or fed fish. *Deiodinase 2b* (*Dio2b*) was also two-fold lower expressed in FDP charr than in fed charr at the end of the experiment (Fig. 5). The lists of top annotated genes that were found differentially expressed in the different comparisons (Additional file 11: Table S9, Additional file 12: Table S10, Additional file 13: Table S11, Additional file 14: Table S12, Additional file 15: Table S13, and Additional file 16: Table S14) were screened for genes that, based on prior knowledge from mammals, are known to be involved in energy homeostasis and control of food intake. Here, the gene nerve growth factor inducible (VGF) was found as a new candidate that may be involved the regulation of appetite and energy metabolism in fish (Fig. 6).

**Table 10:** Differentially expressed candidate genes involved in food intake control and seasonality

			T_1__Fed vs T_0_		T_1__FDP vs T_0_		T_1__FDP vs T_1__Fed	
Gene Name	ContigID	Accession No.	LOG FC	*P* value	LOG FC	*P* value	LOG FC	*P* value
*Apelin receptor A*	Fishapp_brain_apja.2.3	NM_001140368.1	0.99	0.003	–	–	–	–
*Cocaine and amphetamine regulated transcript*	Fishapp_brain_contig_33002	NM_001146680.1	−0.66	< 0.001	−0.33	0.006	–	–
*Cholecystokinin*	Fishapp_brain_contig_18503	NM_001139522.1	–	–	–	–	–	–
	Fishapp_brain_contig_21023		–	–	–	–	–	–
	Fishapp_brain_contig_17948		–	–	−0.37	0.003	–	–
*Corticotropin-releasing factor*	Fishapp_brain_contig_16188	NM_001124627.1	–	–	− 0.55	0.008	–	–
*Deiodinase 2b*	Fishapp_brain_contig_18436	NM_001124268.1	−0.60	0.003	−1.79	< 0.001	−1.19	< 0.001
	Fishapp_brain_contig_15175		−1.21	> 0.001	−2.02	< 0.001	−0.81	< 0.001
*Insulin like growth factor 1*	Fishapp_brain_IGF1	GU933431.1	–	–	−0.81	0.003	–	–
*Leptin*	Fishapp_brain_lepb1	JX131305.1	1.701	< 0.001	1.37	< 0.001	–	–
*Neuropeptide Y*	Fishapp_brain_npy	NM_001146681.1	−0.55	< 0.001	–	–	–	–
*Proopiomelanocortin*	Fishapp_brain_contig_04399	XM_024143555.1	–	–	5.99	0.001	–	–
*Tachykinin 1*	Fishapp_brain_contig_09262	XM_023974799.1	–	–	−0.51	0.002	–	–

**Genes found in the transcriptome, but no difference in expression:**

*Apelin*, *Agouti related peptide*, *Arginine vasotocin*, *Galanin*, *Melanin concentrating hormone*, *Peptide YY*, *Thyroid releasing hormone*

**Genes searched, but not present in the transcriptome:**

*Ghrelin*, *Kisspeptin*, *Leptin receptor*, *Melanocortin receptor 4*, *Obestatin*, *Octadecaneuropeptide*, *Orexin*, *Pituitary adenylate cyclase-activating polypeptide*, *Prolactin releasing peptide*, *Secretoneurin*

### Data release

Our raw RNA-seq data are available in the SRA database [24] under accession number SRP151983. In addition, we specifically developed an interface for the present de novo transcriptome. The entire dataset can be browsed at the following URL: http://ngspipelines2.toulouse.inra.fr:9002/ the main menu enables the user to enter the contigs, SNPs and download sections. The contig section main page displays three blocks providing (1) statistics upon the assembly and the annotations, (2) information and access to statistical analysis tools (digital differential display, Venn diagram) on the library and (3) a table that displays data of the contigs that are of the user’s strongest interest. Once a contig has been selected, the user can view its general annotation including Gene Ontology, check its sequence for possible open reading frames, visualize the annotation location in the “jbrowse view” and scan the alignment coverage along the contig for each library. All these features are accessible through the menu located at the top of the page. The SNP section has also a main page presenting some statistics and the favourite table. Once a variable locus is selected, the user can access general information such as the list of alleles and the flanking sequences, allele information with the allele count graph and table for each library and the corresponding functional annotation.

## Discussion

### Feed deprived and fed charr showed a diverging development of weight and condition

The strong increase in *K* and body mass observed over the 4 weeks period in the fed charr (Fig. 1) was consistent with the high food intake and growth of anadromous Arctic charr during summer [17, 25]. The FDP charr underwent a strong mobilization of energy reserves during the experiment resulting in a markedly lower *K* and body mass in FDP charr than in fed charr at T_1_ (Fig. 1). This effect of treatment was also evident in the 5 fish from each treatment sampled for transcriptome analyses.

### A reliable de novo brain transcriptome

Out of the 49829 generated contigs, more than 85% were annotated, mainly based on Atlantic salmon (Fig. 2). This was expected as Atlantic salmon was phylogenetically the closest related species with a sequenced genome at the time the charr de novo transcriptome was built (spring 2016) [26]. Besides other teleost species contributing to the annotation, *Homo sapiens* also gave hits: the human genome is well characterized and might have brought annotations of genes that, so far, have only been described in humans.

Even if it is known for its complex transcriptomic signature, the brain alone cannot be representative of the entire diversity of the Arctic charr transcriptome. Thus, a completeness of 67% (BUSCO analysis) confirmed that the assembly produced correct contigs. Indeed other fish transcriptome de novo assemblies brought results in the same order of magnitude with 70.2% completeness for the gut tench (*Tinca tinca*) [27] and 64% for 4 combined tissues of the noble crayfish (*Astacus astacus*) [28]. In addition, (1) the comparison to the phylogenetically close Atlantic salmon reference proteome, (2) the calculation of the realignment rates and (3) the mapping of an Arctic charr public raw data using a de novo assembly from short read RNA-seq data on our dataset also confirmed a high completeness of the built transcriptome.

### Global brain gene expression patterns differ over time while feed deprivation has a moderate impact

The correlation heatmap highlighted that charr brain transcriptomes from T_0_ clustered together and were markedly different from the ones sampled at T_1_. Samples of the fed and FDP charr at T_1_ did not cluster in accordance with the treatment group. These results stand in contrast with the strong divergence in *K* and weight between the fed and FDP charr. However, feed deprivation may only have affected very specific processes in the brain transcriptome leaving the overall expression pattern less strongly affected.

Seasonal changes alone (including the increased water temperature), or in combination with feed deprivation, induced a high number of differing contigs from start to end of the experiment, compared to the minor differences observed between the treatment groups at the endpoint. This pattern depicts strong shifts in the charr brain gene expression over a 4 weeks period during summer, which occurred regardless of feeding regime (Fig. 4). The results underline that seasonal processes, including changes in water temperature and summer growth, have much stronger effects on the brain transcriptome in the charr than 1 month of feed deprivation during the feeding season. However, the two times higher number of differentially expressed contigs over time in the feed deprived charr than in the fed conspecifics may suggest an enhancement of seasonal differences by feed deprivation (Table 2). The large changes seen over time correspond with an earlier transcriptome study on different Arctic charr morphs, where time, in that case during early development, was the most important factor for differentially expressed transcripts [29].

In addition, the Up-set graphs (Fig.4) showed that a larger fraction of both up- and down-regulated contigs differed only in response to feed deprivation. On the other hand, most of the differences found in the fed fish were at the same time found in the FDP charr. These shared differences over time depict robust seasonal processes that remain unaffected by feed availability.

### Food deprivation partially offsets increase in brain metabolism during summer growth

#### Feeding specific differences over time

Up-regulated contigs over time denoted foremost oxygen transport (genes encoding for several haemoglobin (Hb) subunits) and protein related biological processes (Table 3). Brain *Hb* mRNA has been found in rodents and humans [30] and it has been suggested that neural haemoglobin may facilitate oxygen transport in neurons [31], but the exact mechanism remains unknown. In addition, it cannot be ruled that the cDNA library may have contained RNA from non-neuronal tissue including blood vessels and veins, e.g. derived from the highly vascularised saccus vasculosus. The metabolic rate of ectotherms is directly linked to ambient temperature. Specifically, the temperature of optimal growth performance of Arctic charr from North-Norway has been shown to be 14 °C [32]. The increase in water temperature by almost 10 °C during our study may have involved an increase in metabolic rate of Arctic charr. Hence, the observed increase in expression of genes encoding oxygen transporters may have occurred in order to meet increased oxygen demands at higher temperatures.

Furthermore, the feeding related terms such as “feeding behaviour” and “negative regulation of appetite” appeared in the list of GO terms of down-regulated contigs. The contig that contributed to these GO terms was the anorexigenic neuropeptide *CART*, thus suggesting an increase in appetite in the fed charr over time (Additional file 4: Table S2). The decrease in *CART* expression is discussed in detail in the paragraph on candidate appetite regulators.

#### Feed deprivation specific differences over time

In the FDP charr, up-regulated contigs were associated with processes such as catabolism, apoptosis, and immune function (Table 7). In contrast, no such trends were found in brain transcriptome analyses of 21-day feed-deprived zebrafish [33]. The finding that contigs relating to apoptosis were increased is puzzling, given the consensus that the brain is well protected from starvation in both mammals [34] and fish [35]. However, in mammals, there is a debate on to what extend feed deprivation may initiate a degeneration of the central nervous system, as different studies have given indication for both absence [36] and presence [37] of autophagy in the brain of feed deprived mice. Further experiments are needed to test whether the observed up-regulation of contigs involved in apoptosis were a sign of neuronal degradation in charr.

Furthermore, we found the GO term “ketone body catabolic process”, comprising the gene encoding 3-oxoacid CoA transferase, to be up-regulated in the feed deprived charr, pointing towards an increase in ketone catabolic activity from start to the end of experiment (Additional file 7: Table S5). This is in line with previous studies on Atlantic salmon and rainbow trout, where ketone bodies were found to serve as an important energy source for the brain when food is absent [38, 39].

In contrast to the fed charr, contigs pertaining to oxygen transport were down-regulated in feed deprived charr (Table 8). This finding is in agreement with the down-regulation of transcripts related to oxygen transport in response to feed deprivation previously seen in Atlantic salmon liver transcriptome [40], rainbow trout liver transcriptome [41] and in zebrafish brain transcriptome [33]. The lower expression of *Hb* in feed deprived charr may be related to a metabolic suppression in FDP fish in order to save energy when feed is absent. Brain metabolic suppression, indicated by a reduction of glucose oxidation has previously been observed in feed deprived rainbow trout [39]. However, these feed deprived rainbow trout showed a decrease in hexokinase and 6-phosphofruktokinase activities in the brain. In contrast, expression of these glycolytic enzymes did not differ between fed and FDP charr in the present study.

#### Differences between fed and feed deprived charr at the end of the experiment

At T_1_, there was an up-regulation of contigs involved in biological processes related to apoptosis by feed deprivation as well as a down-regulation of biological processes related to oxygen transport (Additional file 9: Table S7, Table 9, Additional file 12: Table S10). These findings further support the possibility that brain metabolic processes may have been partly impaired by feed deprivation, as the changes were both visible over time and between the fed and FDP fish at the end of the experiment.

#### Differences over time regardless of feeding regime

Over time, there was an up-regulation of contigs involved in biological processes such as DNA replication, RNA metabolism, response to steroid hormones and immune response (Table 5). This may indicate that basic cell metabolic processes such as cell proliferation and neuronal development were positively affected by seasonal growth and temperature. At the same time, there was a down-regulation of contigs affecting biological processes such as ion transport, protein related processes and wnt signalling from start to end of the experiment (Additional file 6: Table S4).

Interestingly, in adult zebrafish, activation and deactivation of wnt signalling in a sequential manner has been shown to accommodate proliferation and differentiation of progenitor cells in the hypothalamus [42]. Furthermore, the finding that wnt signalling in the ARC was stimulated by leptin in mouse [43] and by both leptin and long photoperiod in the seasonal Djungarian hamster (*Phodopus sungorus*) [44] have triggered a discussion for a role of hypothalamic wnt signalling in the seasonal control of energy balance [45]. Yet, we only found differences in expression for *WNT4*, and the question of a possible seasonal related function of wnt signalling in charr requires further study.

Taken together, the results from the GO analyses indicate that the enforced energy perturbation by feed deprivation may have affected several metabolic processes in the brain at the mRNA level. Yet, most elementary biological processes, including cell division processes and immune responses, differed similarly over time in both fed and feed deprived charr.

### Effect of feed deprivation on candidate genes involved in the regulation of appetite and energy metabolism in fish

#### Expression patterns of known anorexigenic and orexigenic neuropeptides do not indicate an amplified hunger signalling in feed deprived charr

Despite the lower weight and *K* in feed deprived charr, expression of central appetite regulators did not reflect the feeding regime of the fish (Table 10). Several genes differed in one or both feeding regimes over time, but none between FDP and fish at T_1_. There was a lower expression of the anorexigenic *CART* at T_1_ in both fed (LogFC = − 0.66) and FDP (LogFC = − 0.33) charr than in fed charr at T_0_ (Table 10). If CART exhibits an anorexigenic function in charr, the lower expression of *CART* in both fed and FDP fish over time points towards a seasonal increase in hunger signalling in both treatment groups, but no response to feed deprivation. This corresponds to the lack of responses in hypothalamic *CART* expression seen in previous long-term feed deprivation studies with rainbow trout (4 months) and Arctic charr (4 weeks) [19, 46]. In contrast, brain *CART* expression decreased in zebrafish after 3 days feed deprivation [47], in Atlantic salmon after 6 days of feed deprivation [48] and in Atlantic cod (*Gadus morhua*) after 7 days feed deprivation [49].

Being described as a potent satiety signal in mammals and several fish species, hypothalamic *POMCA* had, unexpectedly, markedly (LogFC = 5.99) higher expression levels in FDP charr at T_1_ than in charr sampled at T_0_. In contrast to the results seen for *CART*, this finding may suggest a decrease in hunger signalling in FDP charr over time. This result corresponds to an increased hypothalamic expression of *POMCA*1 and *POMCB* in rainbow trout after 4 months of feed deprivation [46]. In another experiment with rainbow trout, hypothalamic *POMCA1* was down-regulated after 28 days of feed deprivation [50]. However, *POMCA* paralogues could not be distinguished in the present study, and a possible subfunctionalisation of these in the charr, which may be reflected in different responses to feed deprivation, cannot be excluded.

Putatively anorexigenic corticotrophin releasing factor (*CRF*) expression was lower in FDP charr at T_1_ than at T_0_ (LogFC − 0.55). Previously, reduced *CRF* brain expression levels were observed in goldfish (*Carassius auratus*) after 7 days of feed deprivation [51] whereas no changes were observed after short (7 days)- and long-term (4 weeks) feed deprivation in charr [19] or after long-term feed deprivation in rainbow trout [46].

In rat, the preprotachykinin 1 (PPT) protein, encoded by the *TAC1* gene, has been shown to be negatively regulated by ghrelin and high fat diets, and hence is assumed to be involved in regulating adiposity in rodents [52]. In goldfish, the post-prandial increase of hypothalamic expression of *μ-PPT* has led to the suggestion that μ-PPT may signal satiety [53]. In the present study, brain *TAC1* expression was lower expressed in FDP charr at T_1_ than at T_0_ (LogFC − 0.51).

The function of locally produced LEP in the brain is still a matter of debate, both in mammals and in fish [54–56], and results from other studies have so far not provided evidence for a role of central LEP in appetite regulation in fish [18, 56]. This study revealed a higher *LEP* expression in both fed (LogFC 1.7) and FDP fish (LogFC 1.3) at T_1_ compared with T_0_ fish. The lack of differences between fed and FDP charr at T_1_, despite a profound difference in condition factor (Fig. 1), may indicate that central leptin expression is not linked to adiposity. On the other hand, *LEPA1* was found to be more highly expressed in the hypothalamus of hyperphagic charr in July than of anorexic charr in May and January [18].

Previous studies have shown a conserved orexigenic function of NPY in fish [12, 57, 58]. *NPY* expression was lower in fed fish at T_1_ compared to T_0_ (LogFC − 0.55) while no difference was seen between FDP andT_0_. As such, based on the brain transcriptome, no hunger signalling by up-regulation of *NPY* could be found in the FDP fish. This finding is in accordance with the lack of responses seen in other feed deprivation studies with in Atlantic cod [49], Atlantic salmon [48], charr [19] and rainbow trout [46].

Apelin is considered another potent orexigenic actor in fish [59, 60]. Our data did not reveal an effect of long-term feed deprivation on apelin expression. Brain expression of apelin receptor (*APJA*), was, however, higher in fed fish at T_1_ than in fed fish at T_0_ (LogFC = 0.99) but not different between FDP and fed fish at T_0_.

In summary, the results of the present study did not show expected responses to feed deprivation in the expression of candidate genes involved in appetite regulation in fish. This result does not necessarily contradict an appetite regulatory role of these actors in this fish. In a previous study with charr, no differences in the hypothalamic expression of *AgRP*, *MC4R*, *CRF*, *NPY*, *CART*, *POMC*s and *LEPR* were seen between fed and 4 weeks feed deprived fish, whereas the expression of, *CART*, *MC4R* and *AgRP* changed in feed deprived upon re-feeding or exposure to feed flavour for 1 or 5 h [19]. This indicates that changes in gene expression are more likely to be seen during transition stages than during steady-state situations.

Such paradoxical results may be interpreted as an adaptation in high-latitude fish to save energy by reducing feed searching behaviour when feed is absent.

#### Genes related to energy metabolism and seasonality

Insulin-like growth factor 1 (IGF1) is key growth regulating hormone in vertebrates, and plasma levels of IGF1 usually correlate positively with growth in fish [61]. Accordingly, it has been shown that plasma IGF1 levels vary proportionally with increases and decreases in feeding rate in Arctic charr [62]. Furthermore, hypothalamic *IGF1* expression was reduced by 1 month feed deprivation resulting in a positive correlation also between hypothalamic *IGF1* expression and *K* of fed and feed deprived fish [19]. In the present study, *IGF1* expression was, as expected, downregulated in feed deprived charr at T_1_ compared to fed charr at T_0_. However, no difference was found in central *IGF1* expression between fed and feed deprived charr at T_1_, despite the huge difference in *K* between feed deprived and fed charr at T_1_ (Fig. 1). This discrepancy in results between studies on Arctic charr may relate to the fact that hypothalamic *IGF1* expression was measured in the former study by Striberny and Jørgensen [19], while whole brain *IGF1* expression was measured in the present study.

Dio2 converts thyroxin (T4) to the biologically active triiodothyronine (T3) which, in turn, is known as an enhancer of several biological processes and exerts pleiotropic functions in the mammalian brain [63]. In mammals and birds, the increase in day length in spring stimulates hypothalamic *Dio2* expression, thereby stimulating a range of processes related to seasonal phenotype transitions, including appetite [64]. Similarly, it was recently shown that brain expression of the paralogue *Dio2b* was elevated in response to an increased day length in Atlantic salmon [65]. We found a significantly lower hypothalamic *Dio2b* expression at T_1_ than at T_0_ in both fed and FDP charr, and a lower expression in FDP charr than in fed charr at T_1_ (LogFC = − 1.19/− 0.81) (Fig. 5). Our findings may be interpreted as a general decline in *Dio2b* expression during late summer, a decline that may have been enhanced by feed deprivation. In support of the latter, in the seasonal Djungarian hamster (*Phodopus sungorus*), hypothalamic *Dio2* expression was reduced in response to fasting induced torpor during summer [66]. Further experiments are needed to characterize the function of Dio2 in seasonal processes, including feeding behaviour, in the highly seasonal Arctic charr.

### Nerve growth factor inducible (VGF) - a novel candidate involved in the control of appetite and energy homeostasis in fish?

Interestingly, we found brain *VGF* (non-acronymic, nerve growth factor inducible) to be up-regulated (LogFC = 0.54) in FDP compared to fed charr at T_1_ (Fig. 6). To the best of our knowledge, VGF has not been linked to energy metabolism and appetite control in fish. In mammals, the *VGF* gene encodes for a 68 kDa protein precursor that is abundantly expressed in the brain, particularly in the hypothalamus. VGF cleaves into several smaller peptides that have been shown to be involved in a multitude of processes including nerve growth upon injury, seasonality, and food intake/energy metabolism [67]. Several studies in rodents have given evidence for a role of VGF in the control of energy metabolism [68–70] and food intake [71]. Targeted deletion of *VGF* produces a lean, small, and hyperactive mouse [69]. In mammals, the function of *VGF* is complex and not entirely understood. For example, 48 h feed deprivation in mice caused in one study an up-regulation of hypothalamic *VGF* expression [69], and down-regulation in another study [70]. In mice, *VGF* derived neuropeptide TLQP-21 increases energy expenditure without affecting expression of *POMC*/*CART* and *AgRP*/*NPY*, suggesting that TLQP-21 exerts its effects downstream of MC4R signalling [72]. Furthermore, in Siberian hamster, ARC *VGF* expression was induced by a decrease in photoperiod [73] and reduced by T_3_ [74], raising evidence that *VGF* is involved in the control of seasonal feeding in this species. We found *Dio2b* expression to be lower in FDP charr than in fed charr at T_1_. This indicates a reduced thyroid hormone action, which based on the results from Siberian hamster, could be underlying the increased *VGF* expression seen in feed deprived charr. The implication of this gene in both appetite regulation and seasonality in mammals makes it of particular interest in the strongly seasonal Arctic charr.

## Conclusion

In conclusion, the general gene expression patterns in brain transcriptome of fed and feed deprived charr displayed strong shifts in expression of contigs involved in basic cell metabolic processes over time, and only minor differences were seen in response to feed deprivation. However, these changes during the charr’s summer growth appeared to be enhanced by feed deprivation, indicated by a higher number of differentially expressed contigs over time in feed deprived than in fed charr. A decrease in the expression of haemoglobin subunits together with an increase in expression of genes involved in apoptosis, revealed from GO analysis, may indicate a negative effect of feed deprivation on brain metabolism. This is also supported by a substantially stronger reduction in *Dio2b* expression in feed deprived than in fed charr from start to end of the experiment. However, the brain is a heterogeneous tissue that consists of many different cell types, including neural and non-neural tissue; consequently, further studies are needed to get a better spatial resolution of where the observed changes occur.

Generally, a note of caution is expressed when measurements only include RNA abundances and not the corresponding protein levels. However, in a recent study in which a combined transcriptomic and proteomic approach were applied it was concluded that transcriptomic analyses indeed can be used to predict protein copy numbers [75]. Despite the marked divergence of body mass and *K*, no clear hunger signalling was found between fed and feed deprived charr, when searching the lists of differentially expressed contigs for anorexigenic and orexigenic candidate genes known to be involved in appetite regulation in fish. Hence, these results illustrate that even during summer the anadromous charr have a vast ability and flexibility to deal with food deprivation.

## Methods

### Ethics statement

Fish handling and euthanasia (see below) was performed by a competent person and in accordance with the European Union Regulations concerning the protection and welfare of experimental animals (European directive 91/492/CCE). The experiment was approved by the Norwegian Committee on Ethics in Animal Experimentation (ID 3630).

### Feed deprivation experiment and sampling of fish

The charr used in the present study were two-year-old immature offspring of the anadromous Hammerfest strain, originating from wild charr caught in 1984 and since then bred at Tromsø Aquaculture Research Station, where the experiment was carried out. Until the start of the experiment they had been held on natural water temperature and light conditions (transparent roof) and fed a commercial Arctic charr feed (Skretting, Stavanger, Norway) *ad libitum* by automatic feeders. On June 25 (T_0_), 2014, 42 fish were anesthetized in Benzocaine (60 ppm) and tattoo-tagged with Alcian Blue staining dye using a Pan Jet needleless injector (Wright Dental, Dundee, UK). Body mass and fork length were measured and the fish were distributed between two 300 L tanks supplied with fresh water. Another 12 fish were dip netted from the stock tank and euthanized by an overdose of Benzocaine (150 ppm). Body mass and fork length were measured. Subsequently, the fish were decapitated and the belly was cut open for sex determination. In order to rule out potential sex-specific differences, only male fish were sampled for RNA-seq. On a total of 5 fish, brains were dissected out and separated into telencephalon, mesencephalon and hypothalamus. Tissues were stored in 1.5 ml Eppendorf tubes containing 1 ml of RNAlater (ThermoFisher Scientific, MA, USA). Samples were kept at 4 °C for 24 h, and then frozen at − 20 °C until RNA extraction.

On the same day, 220 fish from the stock tank were distributed amongst the two tanks in which the tattooed fish had been placed (130 fish per tank). From then on, the fish in one tank were fed (Fed) with the same commercial feed as before. Fed fish were fed two main meals at 08.00 AM and 3.00 PM by automatic feeders and in addition, between the main meals to ensure excess feed availability. The fish in the other tank were feed deprived (FDP) until the end of the experiment. All fish were held at simulated natural photoperiod (69 °N), which was 24 h light at that time of the year, and both experimental tanks were supplied with fresh water provided by a flow-through system. Ambient natural water temperature was 4.5 °C at the start and 13.5 °C at the end of the experiment. On July 23 (T_1_), 12 fish from each group were euthanized, from which 5 males were measured and sampled as described above. Finally, the tagged fish were anesthetized and measured for body mass and length.

The high number of fish in each treatment group compared to the number of fish sampled was justified by the need to avoid formation of social hierarchies in the fed group. The tagged fish were included in order to monitor the body mass and condition factor development of the fish in the two treatment groups. Fulton’s condition factor (*K*) was calculated according to Ricker (1975): *K* = (W × L^− 3^) × 100, where W is body mass in g, and L is fork length in cm.

### Sample preparation

Tissues were disrupted using TissueLyser II (QIAGEN, Hilden, Germany), and RNA was extracted using the RNeasy Plus Universal Mini Kit (QIAGEN) according to the manufacturer’s protocol. Concentration and purity of RNA were assessed using NanoDrop ND2000c (ThermoFisher Scientific, MA, USA) and when the 260/280 or 260/230 absorbance ratio was below the quality threshold (1.8), samples were further purified using ethanol precipitation. Genomic DNA was removed by treating the RNA with Ambion TURBO DNA-free™ Kit (Life Technologies, CA, USA). In order to obtain a representative view of the main brain areas that have been shown to be involved in central appetite control, 3 μg of RNA of each brain compartment (telencephalon, mesencephalon and hypothalamus) were pooled resulting in a total of 9 μg RNA per brain and individual. Finally, the overall quality of RNA samples was assessed using Bio-rad Experion Bioanalyzer (Bio-rad, CA, USA) and the RQ ranged from 8.7–9.8 indicating high quality of all samples. We did not observe any clustering in MDS plot that would reflect any potential technical variation (Additional file 2: Figure S2). Samples were then shipped on dry-ice to the GenoToul sequencing platform, Toulouse, France for RNA-seq.

### cDNA library construction and paired-end RNA-seq

RNA preparation and sequencing were performed at the GenoToul sequencing platform, Toulouse, France. Fifteen RNA libraries were prepared using the TruSeq RNA sample preparation Kit (Illumina, Hayward, CA, USA), involving the following steps. Poly-As containing mRNA were isolated from 3 μg of total RNA. The mRNA was then chemically fragmented. The cleaved RNA fragments were reverse transcribed into first stranded cDNA using random primers, and second strand cDNA was then synthesized. Adaptors were ligated to the end-repaired cDNA, which contributed to fragment selection after the PCR enrichment step. Each library quality was validated measuring sample concentration and fragment size on an Agilent High Sensitivity DNA chip. Sequence hybridization to the flow cell and cluster generation was achieved using a cBot system and the cluster generation kit (Illumina, Hayward, USA). Hundred base pair fragments were sequenced in paired-end for the 15 samples. The samples were multiplexed and Sequenced By Synthesis (SBS) on an a single lane of the eight-lane flow cell of an Illumina HiSeq 2500 sequencer The sequencing lane of the flow cell was screened by a camera, driven by the HiSeq Control Software. Image correction and base calling was performed using the Real Time Analysis (RTA) software.

### General statistics, data assembly and annotation

#### Testing for differences in body weight and *K*

Data for weight and *K* were not normally distributed and therefore, statistical testing was conducted on LOG-transformed data. Changes over time were tested using a repeated measures ANOVA. When differences were found, a pairwise comparison applying Bonferroni correction for multiple comparisons was used to determine main effects. Differences between treatment groups were tested using a 2-sample t-test. All statistical testing was done with SYSTAT 13 and figures were drawn using SigmaPlot 13 (both Systat Software, CA, USA). The significance level was set to *p* < 0.05.

#### Sequencing data

##### RNA-Seq data assembly, annotation and quality assessment

Read quality was checked within the ng6 environment [76] using fastQC [77] and Burrows-Wheeler Aligner BWA [78] to search for contamination. The reads were assembled with the Drap pipeline (version 1.7) [79]. The individual sample assemblies were performed with runDrap using Oases with kmers 25, 31, 37, 43, 49, 55, 61, 65, 69. The individual contig files filtered by FPKM (Fragments Per Kilobase per Million mapped reads) over one were then merged with runMeta and filtered again by FPKM over one to produce the reference contig set.

Contigs were annotated searching sequence homologies against the following Ensembl protein databases: blastx [80] *Danio rerio*, *Gadus morhua*, *Oreochromis niloticus*, *Oryzias latipes*, *Takifugu rubripes*, *Tetraodon nigroviridis*, *Xiphophorus maculatus*, refseq_rna blastn [81]; swissprot blastp [82]; unigene_Takifugu_rubripes.9 (blastn); unigene_Oryzias_latipes.30 (blastn); unigene_Danio_rerio.126 (blastn); NCBI Arctic char ESTs (blastn); the contigs (blastn). Repeats were identified with repeatMasker [83] (version open-4-0-3, with standard parameters) using Repbase database [84]. The GO annotations were extracted from InterproScan [85] (May 2015 version) [86]. The best SwissProt, RefSeq, or *Salmo salar* NCBI ESTs hit result was used to classify species by best hits contribution.

Different approaches were used to verify the quality of the built contigs. First, the contigs were processed with BUSCO V2 [87] to verify the number of actinopterygii_odb9 reference genes found and their reconstruction state (partial or complete). Then the *Salmo salar* protein sequences made available by the NCBI (GCF_000233375.1_ICSASG_v2_protein.faa) were aligned with BLAT (standard parameter, version 34) on the 6 frames translated contigs [88]. The alignment was filtered to retain only hits with at least 80% identity and 80% coverage giving the size of the set of well-reconstructed contigs. Finally, the assembly was validated by (1) verifying the realignment rate of the reads of each individual sample on the contigs, (2) mapping the reads of a charr gill transcriptome sequenced in 100 bp paired-end (SRA accession: SRX314607) [89] on the contigs. The Arctic charr genome [23] was released after we had completed our de novo transcriptome and related data analyses. Still, in order to further validate the data set, *S. alpinus* proteins and RNAs (NCBI ID: 12179) were aligned to the set of generated contigs using blat v. 35 × 1 with respectively -t = dnax -q = prot and standard parameters. The best blast hit was extracted in both cases. The alignments were filtered keeping only hits with at least 80% identity and 80% coverage.

##### Polymorphism: SNP and microsatellite search

Reads were aligned to the contigs with bwa mem [78]. They were deduplicated with samtools rmdup, then GATK (Version 3.0–0-g6bad1c6) base quality score recalibration was applied [90]. Indel realignment, SNP and INDEL discovery were performed with HaplotypeCaller using standard hard filtering parameters according to GATK Best Practices recommendations [91, 92]. Indels and SNP were independently filtered; 3 per window of 18b with a minimal quality of 30. The micro-satellites discovery was conducted using Tandem Repeats Finder Version 4.04 [89] using the following parameters: 2, 7, 7, 80, 10, 50, 500 -f -d –m ^2^. These analyses will not be interpreted in the current study but were meant to be made accessible on our de novo transcriptome interface, see data mining interface section for URL and details.

#### Differential expression of contigs

##### Patterns of gene expression

Data exploration and gene expression analyses were performed using various packages implemented in R version 3.3.1 (2016-06-21). A sample correlation heatmap based on Pearson’s coefficient of correlation was drawn with pheatmap. The number of reads counts per contig was retrieved and each sample normalized, accounting for compositional differences between the libraries (calcNormFactors function EdgeR).

Paired comparisons of treatments groups were performed in EdgeR package version 3.8.6 [93] according to the users’ guide procedure. We identified differentially expressed contigs using a general linear model and a quasi-likelihood F-test, and correcting for false discovery rate (corrected Benjamini and Hochberg *p* < 0.05). The following contrasts were made (1) Fed fish at T_1_ with T_0_ fish (T_1__Fed versus T_0_),feed deprived fish at T_1_ with T_0_ fish (T_1__FDP versus T_0_) and feed deprived charr at T_1_ versus fed charr at T_1_ (T_1__ FDP versus T_1__Fed). Only contigs with a logFC greater than 0.5 and smaller − 0.5 were kept for a further comparison using Venn diagrams and GO enrichment analyses. Up-set graphs [94] were drawn using UpsetR to visualize unshared and shared lists of up- and down-regulated contigs between all three comparisons (total of 6 lists). Chi-square tests were run to test whether there was a relationship of feeding regime and proportions of uniquely versus shared up- and downregulated contigs (2 × 2 contingency tables) of over-time-comparisons. Lists of up- and down-regulated contigs of the T_1__Fed versus T_0_ and T_1__FDP versus T_0_, comparisons were further compared in the JVenn interface [95] in order to identify (1) contigs that were differentially expressed only in fed charr, (2) contigs that were only differentially expressed in FDP charr and (3) contigs that differed over time regardless of diet, i.e. a seasonal effect independent of the feeding regime (the shared portion of the Venn diagram). Resulting Venn diagrams where drawn in Venn Diagram Plotter [96] (Additional file 1: Figure S1). All lists of differentially expressed genes generated from the initial EdgeR comparison (no log fold change cut-off) were searched for candidate genes known to be involved in appetite regulation and energy metabolism.

##### GO enrichment

Gene ontology (GO) term enrichment was obtained using the TopGo package [97]. It consisted of the identification of terms that host more differentially expressed contigs than expected by chance in a specific comparison. Enrichment of terms by differentially expressed contigs was assessed using Fisher’s exact test (*p* < 0.05). This analysis focused on Biological Processes. Only terms that were enriched by 3 or more contigs were presented in the results.

Each of the three gene lists generated by Venn diagrams (Additional file 1: Figure S1) were investigated for GO enrichment, and lists of up- and down-regulated contigs were analyzed separately. Finally, GO enrichment analysis was conducted with the lists of up- and down-regulated contigs of the endpoint comparison.

#### Data mining interface

The assembled contigs were annotated using the RNA-seq de novo ngs-pipelines processing chain [98] and the results have been uploaded to a web-based user interface build upon biomart [99].

## Additional files

Additional file 1: **Figure S1.** Venn diagrams comparing up- and down-regulated contigs over time between the two treatments: T_1__Fed versus T_0_ and T_1__FDP versus T_0_ (FDR < 0.05. LogFC cut-off 0.5/− 0.5). Yellow: contigs uniquely differentially expressed in T_1__Fed versus T_0_ comparison (input for GO enrichment Table 3 and Table 4, Additional file 3: Table S1, Additional file 4: Table S2), blue: contigs uniquely differentially expressed in T_1__FDP versus T_0_ comparison (input for GO enrichment Table 7 and Table 8, Additional file 7: Table S5, Additional file 8: Table S6). White: contigs that were found to be differentially expressed over time regardless of feeding regime (input for GO enrichment Table 5 and Table 6, Additional file 5: Table S3, Additional file 6: Table S4). (JPG 67 kb)

Additional file 2: **Figure S2.** MDS Plot of the 15 samples. T0 1–5: T0 charr, T1 6–10: T_1__Fed charr, T1 11–15: T_1__FDP charr. (EPS 6 kb)

Additional file 3: **Table S1.** Biological processes enriched by up-regulated contigs only found in Fed versus T_0_ (see Venn diagram Additional file 1: Figure S1). Terms sorted by the number of contributing contigs. (DOCX 19 kb)

Additional file 4: **Table S2.** Biological processes enriched by down-regulated contigs only found in Fed versus T_0_ (see Venn diagram Additional file 1: Figure S1) Terms sorted by the number of contributing contigs. (DOCX 24 kb)

Additional file 5: **Table S3.** Biological processes enriched by up-regulated contigs found in Fed versus T_0_ and FDP versus T_0_ (see Venn diagram Additional file 1: Figure S1). Terms sorted by the number of contributing contigs. (DOCX 28 kb)

Additional file 6: **Table S4.** Biological processes enriched by down-regulated contigs found in Fed versus T_0_ and FDP versus T_0_ (see Venn diagram Additional file 1: Figure S1). Terms sorted by the number of contributing contigs. (DOCX 22 kb)

Additional file 7: **Table S5.** Biological processes enriched by up-regulated contigs only found in FDP versus T_0_ (see Venn diagram Additional file 1: Figure S1). Terms sorted by the number of contributing contigs. (DOCX 23 kb)

Additional file 8: **Table S6.** Biological processes enriched by down-regulated contigs only found FDP versus T_0_ (see Venn diagram Additional file 1: Figure S1). Terms sorted by the number of contributing contigs. (DOCX 25 kb)

Additional file 9: **Table S7.** Biological processes enriched by up-regulated contigs comparing feed deprived versus fed charr at end of experiment. Terms sorted by the number of contributing contigs. (DOCX 22 kb)

Additional file 10: **Table S8.** Biological processes enriched by down-regulated contigs comparing feed deprived versus fed charr at end of experiment. Terms sorted by the number of contributing contigs. (DOCX 21 kb)

Additional file 11: **Table S9.** FDP versus Fed Top annotated up-regulated contigs. LogFC = Log fold change, logCPM = log counts per million, F = F statistic, FDR = false discovery rate. (XLSX 10 kb)

Additional file 12: **Table S10.** FDP versus Fed Top annotated down-regulated contigs. LogFC = Log fold change, logCPM = log counts per million, F = F statistic, FDR = false discovery rate. (XLSX 11 kb)

Additional file 13: **Table S11.** T_1__Fed versus T_0_ Top annotated up-regulated contigs. LogFC = Log fold change, logCPM = log counts per million, F = F statistic, FDR = false discovery rate. (XLSX 13 kb)

Additional file 14: **Table S12.** T_1__Fed versus T_0_ Top annotated down-regulated contigs. LogFC = Log fold change, logCPM = log counts per million, F = F statistic, FDR = false discovery rate. (XLSX 12 kb)

Additional file 15: **Table S13.** T_1__FDP versus T_0_ Top annotated up-regulated contigs. LogFC = Log fold change, logCPM = log counts per million, F = F statistic, FDR = false discovery rate. (XLSX 12 kb)

Additional file 16: **Table S14.** T_1__FDP versus T_0_ Top annotated down-regulated contigs. LogFC = Log fold change, logCPM = log counts per million, F = F statistic, FDR = false discovery rate. (XLSX 12 kb)

## Data Availability

The raw RNA-seq data generated and analysed during the current study are available in the SRA database, https://www.ncbi.nlm.nih.gov/sra under accession number SRP151983. In addition, we specifically developed an interface for this transcriptome. The entire dataset can be browsed at the following URL: http://ngspipelines2.toulouse.inra.fr:9002/.

## Abbreviations

AgRP: Agouti-related peptide
APJA: Apelin receptor
ARC: Arcuate nucleus
CART: Cocaine-and amphetamine regulated transcript
CRF: Corticotrophin releasing factor
DIO2: Deiodinase 2
FDP: Feed deprived
FDR: False discovery rate
FPKM: Fragments Per Kilobase Million
GO: Gene ontology
Hb: Haemoglobin
IGF1: Insulin-like growth factor 1
K: Fulton’s condition factor
LEP: Leptin
LEPR: Leptin receptor
LogFC: Log fold change
MC4R: Melanocortin receptor 4
MCR: Melanocortin receptor
NPY: Neuropeptide Y
POMC: Proopiomelanocortin
PPT: Preprotachykinin
*VGF*: Nerve growth factor inducible
α-MSH: α-melanocyte-stimulating hormone
