# Supplementary figures and images for: Arctic charr brain transcriptome strongly affected by summer seasonal growth but only subtly by feed deprivation
Source: BMC Genomics. 2019 Jun 27;20:529. doi: 10.1186/s12864-019-5874-z (PMC6598377; doi:10.1186/s12864-019-5874-z)

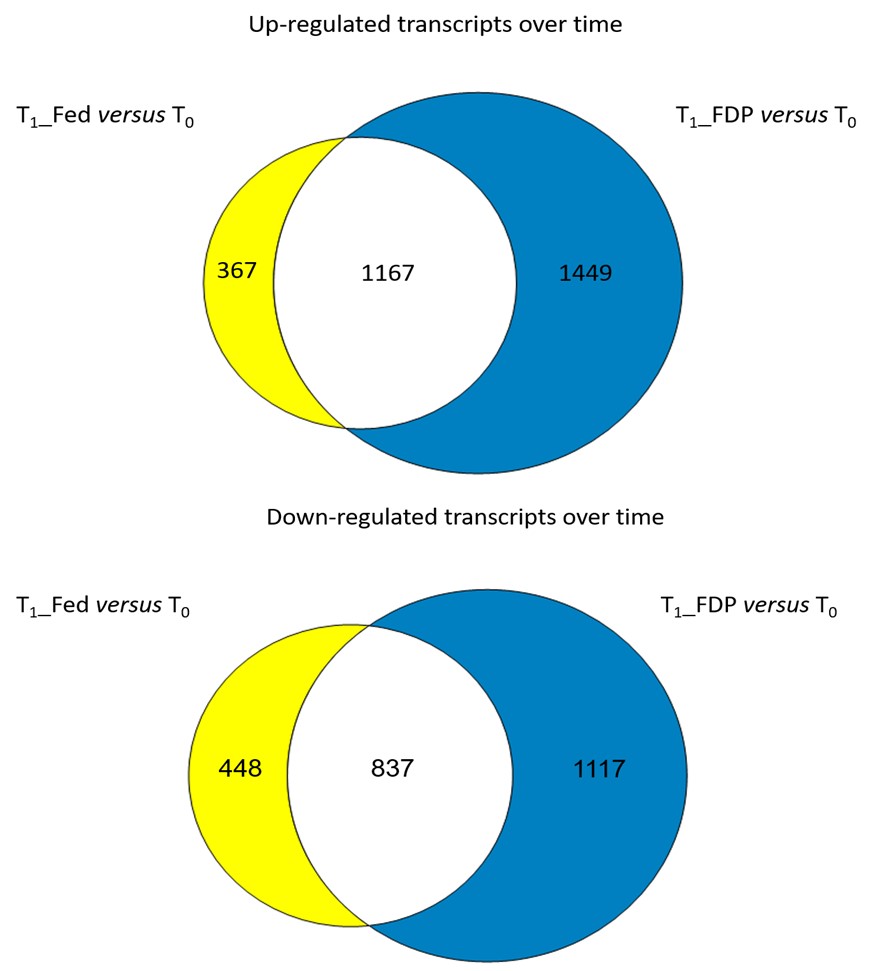

Supplement: Supplementary file 1 — Figure S1. Venn diagrams comparing up- and down-regulated contigs over time between the two treatments: T1_Fed versus T0 and T1_FDP versus T0 (FDR < 0.05. LogFC cut-off 0.5/− 0.5). Yellow: contigs uniquely differentially expressed in T1_Fed versus T0 comparison (input for GO enrichment Table 3 and Table 4, Additional file 3: Table S1, Additional file 4: Table S2), blue: contigs uniquely differentially expressed in T1_FDP versus T0 comparison (input for GO enrichment Table 7 and Table 8, Additional file 7: Table S5, Additional file 8: Table S6). White: contigs that were found to be differentially expressed over time regardless of feeding regime (input for GO enrichment Table 5 and Table 6, Additional file 5: Table S3, Additional file 6: Table S4). (JPG 67 kb) [file 12864_2019_5874_MOESM1_ESM.jpg]
