# Supplementary material for: Arctic charr brain transcriptome strongly affected by summer seasonal growth but only subtly by feed deprivation
Source: BMC Genomics. 2019 Jun 27;20:529. doi: 10.1186/s12864-019-5874-z (PMC6598377; doi:10.1186/s12864-019-5874-z)
Supplement: Supplementary file 3 — Table S1. Biological processes enriched by up-regulated contigs only found in Fed versus T0 (see Venn diagram Additional file 1: Figure S1). Terms sorted by the number of contributing contigs. (DOCX 19 kb) [file 12864_2019_5874_MOESM3_ESM.docx]

**Table S1** Biological processes enriched by up-regulated contigs only found in Fed versus T_0_ (see Venn diagram Supplementary Figure S1). Terms sorted by the number of contributing contigs.

| **GO.ID** | **Term** | **Annotated** | **Significant** | **Expected** | **p-value** |
| --- | --- | --- | --- | --- | --- |
| GO:0006810 | Transport | 1483 | 25 | 14.54 | 0.0026 |
| GO:0051234 | establishment of localization | 1484 | 25 | 14.55 | 0.0026 |
| GO:0051179 | Localization | 1501 | 25 | 14.71 | 0.0031 |
| GO:0044765 | single-organism transport | 883 | 19 | 8.66 | 0.0006 |
| GO:1902578 | single-organism localization | 893 | 19 | 8.75 | 0.0007 |
| GO:0015669 | gas transport | 16 | 10 | 0.16 | 3.20E-17 |
| GO:0015671 | oxygen transport | 16 | 10 | 0.16 | 3.20E-17 |
| GO:0006457 | protein folding | 104 | 5 | 1.02 | 0.0034 |
| GO:0003333 | amino acid transmembrane transport | 15 | 2 | 0.15 | 0.0092 |
| GO:0006865 | amino acid transport | 15 | 2 | 0.15 | 0.0092 |
| GO:1903825 | organic acid transmembrane transport | 15 | 2 | 0.15 | 0.0092 |
| GO:0098656 | anion transmembrane transport | 16 | 2 | 0.16 | 0.0104 |
| GO:0015849 | organic acid transport | 27 | 2 | 0.26 | 0.0284 |
| GO:0046942 | carboxylic acid transport | 27 | 2 | 0.26 | 0.0284 |
| GO:0006414 | translational elongation | 30 | 2 | 0.29 | 0.0345 |
| GO:0051258 | protein polymerization | 36 | 2 | 0.35 | 0.0482 |
| GO:0071705 | nitrogen compound transport | 36 | 2 | 0.35 | 0.0482 |
| GO:0009249 | protein lipoylation | 1 | 1 | 0.01 | 0.0098 |
| GO:0018065 | protein-cofactor linkage | 3 | 1 | 0.03 | 0.0291 |
| GO:0006284 | base-excision repair | 4 | 1 | 0.04 | 0.0386 |
| GO:0046836 | glycolipid transport | 4 | 1 | 0.04 | 0.0386 |
| GO:0006555 | methionine metabolic process | 5 | 1 | 0.05 | 0.0481 |
| GO:0009086 | methionine biosynthetic process | 5 | 1 | 0.05 | 0.0481 |
| GO:0019509 | L-methionine biosynthetic process from methylthioadenosine | 5 | 1 | 0.05 | 0.0481 |
| GO:0043102 | amino acid salvage | 5 | 1 | 0.05 | 0.0481 |
| GO:0046168 | glycerol-3-phosphate catabolic process | 5 | 1 | 0.05 | 0.0481 |
| GO:0071265 | L-methionine biosynthetic process | 5 | 1 | 0.05 | 0.0481 |
| GO:0071267 | L-methionine salvage | 5 | 1 | 0.05 | 0.0481 |
