# Supplementary material for: Arctic charr brain transcriptome strongly affected by summer seasonal growth but only subtly by feed deprivation
Source: BMC Genomics. 2019 Jun 27;20:529. doi: 10.1186/s12864-019-5874-z (PMC6598377; doi:10.1186/s12864-019-5874-z)
Supplement: Supplementary file 4 — Table S2. Biological processes enriched by down-regulated contigs only found in Fed versus T0 (see Venn diagram Additional file 1: Figure S1) Terms sorted by the number of contributing contigs. (DOCX 24 kb) [file 12864_2019_5874_MOESM4_ESM.docx]

**Table S2** Biological processes enriched by down-regulated contigs only found in Fed versus T_0_ (see Venn diagram Supplementary Figure S1) Terms sorted by the number of contributing contigs.

| **GO.ID** | **Term** | **Annotated** | **Significant** | **Expected** | **p-value** |
| --- | --- | --- | --- | --- | --- |
| GO:0044765 | single-organism transport | 883 | 11 | 5.77 | 0.02405 |
| GO:1902578 | single-organism localization | 893 | 11 | 5.84 | 0.02593 |
| GO:0006811 | ion transport | 532 | 10 | 3.48 | 0.0019 |
| GO:0006461 | protein complex assembly | 173 | 4 | 1.13 | 0.0258 |
| GO:0070271 | protein complex biogenesis | 173 | 4 | 1.13 | 0.0258 |
| GO:0065003 | macromolecular complex assembly | 189 | 4 | 1.24 | 0.03419 |
| GO:0071822 | protein complex subunit organization | 189 | 4 | 1.24 | 0.03419 |
| GO:0009966 | regulation of signal transduction | 212 | 4 | 1.39 | 0.04877 |
| GO:0010646 | regulation of cell communication | 213 | 4 | 1.39 | 0.04947 |
| GO:0023051 | regulation of signalling | 213 | 4 | 1.39 | 0.04947 |
| GO:0008272 | sulfate transport | 3 | 2 | 0.02 | 0.00012 |
| GO:0072348 | sulfur compound transport | 3 | 2 | 0.02 | 0.00012 |
| GO:0015698 | inorganic anion transport | 31 | 2 | 0.2 | 0.01723 |
| GO:0048522 | positive regulation of cellular process | 47 | 2 | 0.31 | 0.03749 |
| GO:0048585 | negative regulation of response to stimulus | 51 | 2 | 0.33 | 0.04351 |
| GO:0051260 | protein homooligomerization | 55 | 2 | 0.36 | 0.04985 |
| GO:0008614 | pyridoxine metabolic process | 2 | 1 | 0.01 | 0.01303 |
| GO:0008615 | pyridoxine biosynthetic process | 2 | 1 | 0.01 | 0.01303 |
| GO:0042816 | vitamin B6 metabolic process | 2 | 1 | 0.01 | 0.01303 |
| GO:0042819 | vitamin B6 biosynthetic process | 2 | 1 | 0.01 | 0.01303 |
| GO:0007172 | signal complex assembly | 3 | 1 | 0.02 | 0.01948 |
| GO:0009110 | vitamin biosynthetic process | 3 | 1 | 0.02 | 0.01948 |
| GO:0042364 | water-soluble vitamin biosynthetic process | 3 | 1 | 0.02 | 0.01948 |
| GO:0001678 | cellular glucose homeostasis | 5 | 1 | 0.03 | 0.03226 |
| GO:0007631 | feeding behaviour | 5 | 1 | 0.03 | 0.03226 |
| GO:0008343 | adult feeding behaviour | 5 | 1 | 0.03 | 0.03226 |
| GO:0009267 | cellular response to starvation | 5 | 1 | 0.03 | 0.03226 |
| GO:0009991 | response to extracellular stimulus | 5 | 1 | 0.03 | 0.03226 |
| GO:0030534 | adult behaviour | 5 | 1 | 0.03 | 0.03226 |
| GO:0031667 | response to nutrient levels | 5 | 1 | 0.03 | 0.03226 |
| GO:0031668 | cellular response to extracellular stimulus | 5 | 1 | 0.03 | 0.03226 |
| GO:0031669 | cellular response to nutrient levels | 5 | 1 | 0.03 | 0.03226 |
| GO:0032094 | response to food | 5 | 1 | 0.03 | 0.03226 |
| GO:0032095 | regulation of response to food | 5 | 1 | 0.03 | 0.03226 |
| GO:0032096 | negative regulation of response to food | 5 | 1 | 0.03 | 0.03226 |
| GO:0032098 | regulation of appetite | 5 | 1 | 0.03 | 0.03226 |
| GO:0032099 | negative regulation of appetite | 5 | 1 | 0.03 | 0.03226 |
| GO:0032101 | regulation of response to external stimulus | 5 | 1 | 0.03 | 0.03226 |
| GO:0032102 | negative regulation of response to external stimulus | 5 | 1 | 0.03 | 0.03226 |
| GO:0032104 | regulation of response to extracellular stimulus | 5 | 1 | 0.03 | 0.03226 |
| GO:0032105 | negative regulation of response to extracellular stimulus | 5 | 1 | 0.03 | 0.03226 |
| GO:0032107 | regulation of response to nutrient levels | 5 | 1 | 0.03 | 0.03226 |
| GO:0032108 | negative regulation of response to nutrients | 5 | 1 | 0.03 | 0.03226 |
| GO:0033500 | carbohydrate homeostasis | 5 | 1 | 0.03 | 0.03226 |
| GO:0042593 | glucose homeostasis | 5 | 1 | 0.03 | 0.03226 |
| GO:0042594 | response to starvation | 5 | 1 | 0.03 | 0.03226 |
| GO:0044708 | single-organism behaviour | 5 | 1 | 0.03 | 0.03226 |
| GO:0071496 | cellular response to external stimulus | 5 | 1 | 0.03 | 0.03226 |
| GO:0000186 | activation of MAPKK activity | 6 | 1 | 0.04 | 0.03859 |
| GO:0006766 | vitamin metabolic process | 6 | 1 | 0.04 | 0.03859 |
| GO:0006767 | water-soluble vitamin metabolic process | 6 | 1 | 0.04 | 0.03859 |
| GO:0007610 | Behaviour | 6 | 1 | 0.04 | 0.03859 |
| GO:0010506 | regulation of autophagy | 6 | 1 | 0.04 | 0.03859 |
| GO:0032147 | activation of protein kinase activity | 6 | 1 | 0.04 | 0.03859 |
| GO:0033674 | positive regulation of kinase activity | 6 | 1 | 0.04 | 0.03859 |
| GO:0043405 | regulation of MAP kinase activity | 6 | 1 | 0.04 | 0.03859 |
| GO:0045860 | positive regulation of protein kinase activity | 6 | 1 | 0.04 | 0.03859 |
| GO:0051347 | positive regulation of transferase activity | 6 | 1 | 0.04 | 0.03859 |
| GO:0000165 | MAPK cascade | 7 | 1 | 0.05 | 0.04488 |
| GO:0001934 | positive regulation of protein phosphorylation | 7 | 1 | 0.05 | 0.04488 |
| GO:0010562 | positive regulation of phosphorus metabolic process | 7 | 1 | 0.05 | 0.04488 |
| GO:0023014 | signal transduction by protein phosphorylation | 7 | 1 | 0.05 | 0.04488 |
| GO:0031329 | regulation of cellular catabolic process | 7 | 1 | 0.05 | 0.04488 |
| GO:0031401 | positive regulation of protein modification process | 7 | 1 | 0.05 | 0.04488 |
| GO:0042327 | positive regulation of phosphorylation | 7 | 1 | 0.05 | 0.04488 |
| GO:0043408 | regulation of MAPK cascade | 7 | 1 | 0.05 | 0.04488 |
| GO:0043410 | positive regulation of MAPK cascade | 7 | 1 | 0.05 | 0.04488 |
| GO:0045937 | positive regulation of phosphate metabolic process | 7 | 1 | 0.05 | 0.04488 |
