# Supplementary material for: Arctic charr brain transcriptome strongly affected by summer seasonal growth but only subtly by feed deprivation
Source: BMC Genomics. 2019 Jun 27;20:529. doi: 10.1186/s12864-019-5874-z (PMC6598377; doi:10.1186/s12864-019-5874-z)
Supplement: Supplementary file 5 — Table S3. Biological processes enriched by up-regulated contigs found in Fed versus T0 and FDP versus T0 (see Venn diagram Additional file 1: Figure S1). Terms sorted by the number of contributing contigs. (DOCX 28 kb) [file 12864_2019_5874_MOESM5_ESM.docx]

**Table S3** Biological processes enriched by up-regulated contigs found in Fed versus T_0_ and FDP versus T_0_ (see Venn diagram Supplementary Figure S1). Terms sorted by the number of contributing contigs.

| **GO.ID** | **Term** | **Annotated** | **Significant** | **Expected** | **p-value** |
| --- | --- | --- | --- | --- | --- |
| GO:0019438 | aromatic compound biosynthetic process | 782 | 28 | 16 | 0.0021 |
| GO:0018130 | heterocycle biosynthetic process | 796 | 28 | 16.28 | 0.0027 |
| GO:1901362 | organic cyclic compound biosynthetic process | 803 | 28 | 16.43 | 0.0031 |
| GO:0034654 | nucleobase-containing compound biosynthetic process | 750 | 25 | 15.34 | 0.0092 |
| GO:0080090 | regulation of primary metabolic process | 622 | 24 | 12.72 | 0.0017 |
| GO:0031323 | regulation of cellular metabolic process | 632 | 24 | 12.93 | 0.0021 |
| GO:0019222 | regulation of metabolic process | 641 | 24 | 13.11 | 0.0025 |
| GO:0019219 | regulation of nucleobase-containing compound metabolic process | 577 | 23 | 11.8 | 0.0014 |
| GO:0051171 | regulation of nitrogen compound metabolic process | 587 | 23 | 12.01 | 0.0017 |
| GO:0060255 | regulation of macromolecule metabolic process | 619 | 23 | 12.66 | 0.0034 |
| GO:0006355 | regulation of transcription, DNA-templated | 568 | 22 | 11.62 | 0.0025 |
| GO:1903506 | regulation of nucleic acid-templated transcription | 568 | 22 | 11.62 | 0.0025 |
| GO:2001141 | regulation of RNA biosynthetic process | 569 | 22 | 11.64 | 0.0026 |
| GO:0051252 | regulation of RNA metabolic process | 572 | 22 | 11.7 | 0.0028 |
| GO:0010556 | regulation of macromolecule biosynthetic process | 580 | 22 | 11.87 | 0.0033 |
| GO:2000112 | regulation of cellular macromolecule biosynthetic process | 580 | 22 | 11.87 | 0.0033 |
| GO:0031326 | regulation of cellular biosynthetic process | 581 | 22 | 11.89 | 0.0034 |
| GO:0009889 | regulation of biosynthetic process | 582 | 22 | 11.91 | 0.0034 |
| GO:0010468 | regulation of gene expression | 583 | 22 | 11.93 | 0.0035 |
| GO:0006351 | transcription, DNA-templated | 630 | 22 | 12.89 | 0.0087 |
| GO:0097659 | nucleic acid-templated transcription | 630 | 22 | 12.89 | 0.0087 |
| GO:0032774 | RNA biosynthetic process | 633 | 22 | 12.95 | 0.0092 |
| GO:0051276 | chromosome organization | 94 | 7 | 1.92 | 0.003 |
| GO:0006325 | chromatin organization | 72 | 6 | 1.47 | 0.0034 |
| GO:0006955 | immune response | 50 | 5 | 1.02 | 0.0034 |
| GO:0002376 | immune system process | 52 | 5 | 1.06 | 0.004 |
| GO:0016571 | histone methylation | 11 | 3 | 0.23 | 0.0012 |
| GO:0018022 | peptidyl-lysine methylation | 11 | 3 | 0.23 | 0.0012 |
| GO:0034968 | histone lysine methylation | 11 | 3 | 0.23 | 0.0012 |
| GO:0018205 | peptidyl-lysine modification | 22 | 3 | 0.45 | 0.0097 |
| GO:0016569 | covalent chromatin modification | 23 | 3 | 0.47 | 0.011 |
| GO:0016570 | histone modification | 23 | 3 | 0.47 | 0.011 |
| GO:0019882 | antigen processing and presentation | 26 | 3 | 0.53 | 0.0154 |
| GO:0006479 | protein methylation | 27 | 3 | 0.55 | 0.0171 |
| GO:0008213 | protein alkylation | 27 | 3 | 0.55 | 0.0171 |
| GO:0033993 | response to lipid | 28 | 3 | 0.57 | 0.0189 |
| GO:0043401 | steroid hormone mediated signaling pathway | 28 | 3 | 0.57 | 0.0189 |
| GO:0048545 | response to steroid hormone | 28 | 3 | 0.57 | 0.0189 |
| GO:0071383 | cellular response to steroid hormone stimulus | 28 | 3 | 0.57 | 0.0189 |
| GO:0071396 | cellular response to lipid | 28 | 3 | 0.57 | 0.0189 |
| GO:0014070 | response to organic cyclic compound | 29 | 3 | 0.59 | 0.0208 |
| GO:0071407 | cellular response to organic cyclic compound | 29 | 3 | 0.59 | 0.0208 |
| GO:0009725 | response to hormone | 31 | 3 | 0.63 | 0.0248 |
| GO:0009755 | hormone-mediated signaling pathway | 31 | 3 | 0.63 | 0.0248 |
| GO:0032870 | cellular response to hormone stimulus | 31 | 3 | 0.63 | 0.0248 |
| GO:0018193 | peptidyl-amino acid modification | 34 | 3 | 0.7 | 0.0316 |
| GO:0006334 | nucleosome assembly | 35 | 3 | 0.72 | 0.0341 |
| GO:0016568 | chromatin modification | 35 | 3 | 0.72 | 0.0341 |
| GO:0031497 | chromatin assembly | 35 | 3 | 0.72 | 0.0341 |
| GO:0034728 | nucleosome organization | 35 | 3 | 0.72 | 0.0341 |
| GO:0006323 | DNA packaging | 36 | 3 | 0.74 | 0.0366 |
| GO:0006333 | chromatin assembly or disassembly | 37 | 3 | 0.76 | 0.0393 |
| GO:0033014 | tetrapyrrole biosynthetic process | 14 | 2 | 0.29 | 0.0322 |
| GO:0007093 | mitotic cell cycle checkpoint | 1 | 1 | 0.02 | 0.0205 |
| GO:0007094 | mitotic spindle assembly checkpoint | 1 | 1 | 0.02 | 0.0205 |
| GO:0009888 | tissue development | 1 | 1 | 0.02 | 0.0205 |
| GO:0009895 | negative regulation of catabolic process | 1 | 1 | 0.02 | 0.0205 |
| GO:0015696 | ammonium transport | 1 | 1 | 0.02 | 0.0205 |
| GO:0031099 | regeneration | 1 | 1 | 0.02 | 0.0205 |
| GO:0031330 | negative regulation of cellular catabolic process | 1 | 1 | 0.02 | 0.0205 |
| GO:0031396 | regulation of protein ubiquitination | 1 | 1 | 0.02 | 0.0205 |
| GO:0031397 | negative regulation of protein ubiquitination | 1 | 1 | 0.02 | 0.0205 |
| GO:0032434 | regulation of proteasomal ubiquitin-dependent catabolic process | 1 | 1 | 0.02 | 0.0205 |
| GO:0032435 | negative regulation of proteasomal ubiquitin dependent protein catabolic process | 1 | 1 | 0.02 | 0.0205 |
| GO:0033046 | negative regulation of sister chromatid segregation | 1 | 1 | 0.02 | 0.0205 |
| GO:0033048 | negative regulation of mitotic sister chromatid segregation | 1 | 1 | 0.02 | 0.0205 |
| GO:0042177 | negative regulation of protein catabolic process | 1 | 1 | 0.02 | 0.0205 |
| GO:0042246 | tissue regeneration | 1 | 1 | 0.02 | 0.0205 |
| GO:0045839 | negative regulation of mitotic nuclear division | 1 | 1 | 0.02 | 0.0205 |
| GO:0045841 | negative regulation of mitotic metaphase/anaphase transition | 1 | 1 | 0.02 | 0.0205 |
| GO:0045861 | negative regulation of proteolysis | 1 | 1 | 0.02 | 0.0205 |
| GO:0045930 | negative regulation of mitotic cell cycle | 1 | 1 | 0.02 | 0.0205 |
| GO:0048589 | developmental growth | 1 | 1 | 0.02 | 0.0205 |
| GO:0051340 | regulation of ligase activity | 1 | 1 | 0.02 | 0.0205 |
| GO:0051352 | negative regulation of ligase activity | 1 | 1 | 0.02 | 0.0205 |
| GO:0051436 | negative regulation of ubiquitin-protein ligase activity involved in mitotic cell cycle | 1 | 1 | 0.02 | 0.0205 |
| GO:0051438 | regulation of ubiquitin-protein transferase activity | 1 | 1 | 0.02 | 0.0205 |
| GO:0051439 | regulation of ubiquitin-protein ligase activity | 1 | 1 | 0.02 | 0.0205 |
| GO:0051444 | negative regulation of ubiquitin-protein transferase activity | 1 | 1 | 0.02 | 0.0205 |
| GO:0051725 | protein de-ADP-ribosylation | 1 | 1 | 0.02 | 0.0205 |
| GO:0051782 | negative regulation of cell division | 1 | 1 | 0.02 | 0.0205 |
| GO:0051784 | negative regulation of nuclear division | 1 | 1 | 0.02 | 0.0205 |
| GO:0051985 | negative regulation of chromosome segregation | 1 | 1 | 0.02 | 0.0205 |
| GO:0061136 | regulation of proteasomal protein catabolic process | 1 | 1 | 0.02 | 0.0205 |
| GO:0071173 | spindle assembly checkpoint | 1 | 1 | 0.02 | 0.0205 |
| GO:0071174 | mitotic spindle checkpoint | 1 | 1 | 0.02 | 0.0205 |
| GO:1901799 | negative regulation of proteasomal protein catabolic process | 1 | 1 | 0.02 | 0.0205 |
| GO:1901988 | negative regulation of cell cycle phase transition | 1 | 1 | 0.02 | 0.0205 |
| GO:1901991 | negative regulation of mitotic cell cycle phase transition | 1 | 1 | 0.02 | 0.0205 |
| GO:1902100 | negative regulation of metaphase/anaphase transition of cell cycle | 1 | 1 | 0.02 | 0.0205 |
| GO:1903050 | regulation of proteolysis involved in cellular protein catabolic process | 1 | 1 | 0.02 | 0.0205 |
| GO:1903051 | negative regulation of proteolysis involed in cellular protein catabolic process | 1 | 1 | 0.02 | 0.0205 |
| GO:1903320 | regulation of protein modification by small protein conjugation or removal | 1 | 1 | 0.02 | 0.0205 |
| GO:1903321 | negative regulation of protein modification by small protein conjugation or removal | 1 | 1 | 0.02 | 0.0205 |
| GO:1903362 | regulation of cellular protein catabolic process | 1 | 1 | 0.02 | 0.0205 |
| GO:1903363 | negative regulation of cellular protein catabolic process | 1 | 1 | 0.02 | 0.0205 |
| GO:1904666 | regulation of ubiquitin protein ligase activity | 1 | 1 | 0.02 | 0.0205 |
| GO:1904667 | negative regulation of ubiquitin protein ligase activity | 1 | 1 | 0.02 | 0.0205 |
| GO:2000816 | negative regulation of mitotic sister chromatid separation | 1 | 1 | 0.02 | 0.0205 |
| GO:2001251 | negative regulation of chromosome organization | 1 | 1 | 0.02 | 0.0205 |
