# Supplementary material for: Arctic charr brain transcriptome strongly affected by summer seasonal growth but only subtly by feed deprivation
Source: BMC Genomics. 2019 Jun 27;20:529. doi: 10.1186/s12864-019-5874-z (PMC6598377; doi:10.1186/s12864-019-5874-z)
Supplement: Supplementary file 6 — Table S4. Biological processes enriched by down-regulated contigs found in Fed versus T0 and FDP versus T0 (see Venn diagram Additional file 1: Figure S1). Terms sorted by the number of contributing contigs. (DOCX 22 kb) [file 12864_2019_5874_MOESM6_ESM.docx]

**Table S4** Biological processes enriched by down-regulated contigs found in Fed versus T_0_ and FDP versus T_0_ (see Venn diagram Supplementary Figure S1). Terms sorted by the number of contributing contigs.

| **GO.ID** | **Term** | **Annotated** | **Significant** | **Expected** | **p-value** |
| --- | --- | --- | --- | --- | --- |
| GO:0044699 | single-organism process | 3629 | 54 | 45.37 | 0.04 |
| GO:0044765 | single-organism transport | 883 | 17 | 11.04 | 0.0443 |
| GO:1902578 | single-organism localization | 893 | 17 | 11.16 | 0.0485 |
| GO:0006811 | ion transport | 532 | 12 | 6.65 | 0.0322 |
| GO:0006470 | protein dephosphorylation | 126 | 6 | 1.58 | 0.0048 |
| GO:0016311 | dephosphorylation | 162 | 6 | 2.03 | 0.0156 |
| GO:0015672 | monovalent inorganic cation transport | 175 | 6 | 2.19 | 0.0219 |
| GO:0006813 | potassium ion transport | 88 | 4 | 1.1 | 0.024 |
| GO:0006457 | protein folding | 104 | 4 | 1.3 | 0.0408 |
| GO:0051258 | protein polymerization | 36 | 3 | 0.45 | 0.01 |
| GO:0006304 | DNA modification | 6 | 2 | 0.08 | 0.0022 |
| GO:0006305 | DNA alkylation | 6 | 2 | 0.08 | 0.0022 |
| GO:0006306 | DNA methylation | 6 | 2 | 0.08 | 0.0022 |
| GO:0044728 | DNA methylation or demethylation | 6 | 2 | 0.08 | 0.0022 |
| GO:0006835 | dicarboxylic acid transport | 9 | 2 | 0.11 | 0.0053 |
| GO:0016055 | Wnt signaling pathway | 11 | 2 | 0.14 | 0.0079 |
| GO:0045892 | negative regulation of transcription. DNA templated | 12 | 2 | 0.15 | 0.0094 |
| GO:0051253 | negative regulation of RNA metabolic process | 12 | 2 | 0.15 | 0.0094 |
| GO:1902679 | negative regulation of RNA biosynthetic synthetic process | 12 | 2 | 0.15 | 0.0094 |
| GO:1903507 | negative regulation of nucleic acid-templated transcription | 12 | 2 | 0.15 | 0.0094 |
| GO:0006071 | glycerol metabolic process | 13 | 2 | 0.16 | 0.011 |
| GO:0019400 | alditol metabolic process | 13 | 2 | 0.16 | 0.011 |
| GO:0045934 | negative regulation of nucleobase-containing compound metabolic process | 16 | 2 | 0.2 | 0.0165 |
| GO:0019751 | polyol metabolic process | 17 | 2 | 0.21 | 0.0186 |
| GO:0010558 | negative regulation of macromolecule biosynthetic process | 19 | 2 | 0.24 | 0.023 |
| GO:2000113 | negative regulation of cellular macromolecule biosynthetic process | 19 | 2 | 0.24 | 0.023 |
| GO:0007205 | protein kinase C-activating G-protein coupled receptor | 20 | 2 | 0.25 | 0.0254 |
| GO:0010629 | negative regulation of gene expression | 20 | 2 | 0.25 | 0.0254 |
| GO:0031327 | negative regulation of cellular biosynthetic process | 20 | 2 | 0.25 | 0.0254 |
| GO:0009890 | negative regulation of biosynthetic process | 21 | 2 | 0.26 | 0.0278 |
| GO:0010605 | negative regulation of macromolecule metabolic process | 22 | 2 | 0.28 | 0.0304 |
| GO:0051172 | negative regulation of nitrogen compound metabolic process | 23 | 2 | 0.29 | 0.033 |
| GO:0006066 | alcohol metabolic process | 25 | 2 | 0.31 | 0.0385 |
| GO:0031324 | negative regulation of cellular metabolic process | 25 | 2 | 0.31 | 0.0385 |
| GO:0009892 | negative regulation of metabolic process | 27 | 2 | 0.34 | 0.0443 |
| GO:0015849 | organic acid transport | 27 | 2 | 0.34 | 0.0443 |
| GO:0046942 | carboxylic acid transport | 27 | 2 | 0.34 | 0.0443 |
| GO:0000087 | mitotic M phase | 1 | 1 | 0.01 | 0.0125 |
| GO:0000279 | M phase | 1 | 1 | 0.01 | 0.0125 |
| GO:0022403 | cell cycle phase | 1 | 1 | 0.01 | 0.0125 |
| GO:0044848 | biological phase | 1 | 1 | 0.01 | 0.0125 |
| GO:0098763 | mitotic cell cycle phase | 1 | 1 | 0.01 | 0.0125 |
| GO:0017182 | peptidyl-diphthamide metabolic process | 2 | 1 | 0.03 | 0.0248 |
| GO:0017183 | peptidyl-diphthamide biosynthetic process | 2 | 1 | 0.03 | 0.0248 |
| GO:0018202 | peptidyl-histidine modification | 2 | 1 | 0.03 | 0.0248 |
| GO:0006189 | *'de novo*' IMP biosynthetic process | 3 | 1 | 0.04 | 0.037 |
| GO:0009186 | deoxyribonucleoside diphosphate metabolic process | 3 | 1 | 0.04 | 0.037 |
| GO:0006359 | regulation of transcription from RNA polymerase III | 4 | 1 | 0.05 | 0.0491 |
| GO:0016480 | negative regulation of transcription from RNA polymerase III | 4 | 1 | 0.05 | 0.0491 |
