# Supplementary material for: Arctic charr brain transcriptome strongly affected by summer seasonal growth but only subtly by feed deprivation
Source: BMC Genomics. 2019 Jun 27;20:529. doi: 10.1186/s12864-019-5874-z (PMC6598377; doi:10.1186/s12864-019-5874-z)
Supplement: Supplementary file 7 — Table S5. Biological processes enriched by up-regulated contigs only found in FDP versus T0 (see Venn diagram Additional file 1: Figure S1). Terms sorted by the number of contributing contigs. (DOCX 23 kb) [file 12864_2019_5874_MOESM7_ESM.docx]

**Table S5** Biological processes enriched by up-regulated contigs only found in FDP versus T_0_ (see Venn diagram Supplementary Figure S1). Terms sorted by the number of contributing contigs.

| **GO.ID** | **Term** | **Annotated** | **Significant** | **Expected** | **p-value** |
| --- | --- | --- | --- | --- | --- |
| GO:0065007 | biological regulation | 2021 | 62 | 47.66 | 0.00925 |
| GO:0050789 | regulation of biological process | 1973 | 61 | 46.53 | 0.00837 |
| GO:0050794 | regulation of cellular process | 1934 | 59 | 45.61 | 0.01308 |
| GO:0006725 | cellular aromatic compound metabolic process | 1375 | 42 | 32.43 | 0.03914 |
| GO:0090304 | nucleic acid metabolic process | 1097 | 35 | 25.87 | 0.03448 |
| GO:0016070 | RNA metabolic process | 884 | 30 | 20.85 | 0.02407 |
| GO:0060255 | regulation of macromolecule metabolic process | 619 | 22 | 14.6 | 0.03315 |
| GO:0080090 | regulation of primary metabolic process | 622 | 22 | 14.67 | 0.03473 |
| GO:0031323 | regulation of cellular metabolic process | 632 | 22 | 14.9 | 0.04041 |
| GO:0019222 | regulation of metabolic process | 641 | 22 | 15.12 | 0.04609 |
| GO:0051252 | regulation of RNA metabolic process | 572 | 20 | 13.49 | 0.04778 |
| GO:0006396 | RNA processing | 189 | 10 | 4.46 | 0.01367 |
| GO:0006955 | immune response | 50 | 7 | 1.18 | 0.00015 |
| GO:0002376 | immune system process | 52 | 7 | 1.23 | 0.0002 |
| GO:0008219 | cell death | 58 | 6 | 1.37 | 0.00231 |
| GO:0016265 | death | 58 | 6 | 1.37 | 0.00231 |
| GO:0010941 | regulation of cell death | 45 | 5 | 1.06 | 0.00391 |
| GO:0042981 | regulation of apoptotic process | 45 | 5 | 1.06 | 0.00391 |
| GO:0043067 | regulation of programmed cell death | 45 | 5 | 1.06 | 0.00391 |
| GO:0006915 | apoptotic process | 57 | 5 | 1.34 | 0.0107 |
| GO:0012501 | programmed cell death | 57 | 5 | 1.34 | 0.0107 |
| GO:0048518 | positive regulation of biological process | 67 | 5 | 1.58 | 0.02048 |
| GO:0006397 | mRNA processing | 68 | 5 | 1.6 | 0.0217 |
| GO:0016071 | mRNA metabolic process | 79 | 5 | 1.86 | 0.03824 |
| GO:0019882 | antigen processing and presentation | 26 | 4 | 0.61 | 0.00297 |
| GO:0051726 | regulation of cell cycle | 40 | 4 | 0.94 | 0.01409 |
| GO:0015074 | DNA integration | 55 | 4 | 1.3 | 0.04013 |
| GO:0010942 | positive regulation of cell death | 13 | 3 | 0.31 | 0.0031 |
| GO:0043065 | positive regulation of apoptotic process | 13 | 3 | 0.31 | 0.0031 |
| GO:0043068 | positive regulation of programmed cell death | 13 | 3 | 0.31 | 0.0031 |
| GO:0007050 | cell cycle arrest | 18 | 3 | 0.42 | 0.0081 |
| GO:0045786 | negative regulation of cell cycle | 21 | 3 | 0.5 | 0.01254 |
| GO:0008380 | RNA splicing | 26 | 3 | 0.61 | 0.0225 |
| GO:0006729 | tetrahydrobiopterin biosynthetic process | 5 | 2 | 0.12 | 0.00528 |
| GO:0046146 | tetrahydrobiopterin metabolic process | 5 | 2 | 0.12 | 0.00528 |
| GO:0006405 | RNA export from nucleus | 7 | 2 | 0.17 | 0.01074 |
| GO:0006406 | mRNA export from nucleus | 7 | 2 | 0.17 | 0.01074 |
| GO:0051028 | mRNA transport | 7 | 2 | 0.17 | 0.01074 |
| GO:0051168 | nuclear export | 7 | 2 | 0.17 | 0.01074 |
| GO:0071166 | ribonucleoprotein complex localization | 7 | 2 | 0.17 | 0.01074 |
| GO:0071426 | ribonucleoprotein complex export from nucleus | 7 | 2 | 0.17 | 0.01074 |
| GO:0071427 | mRNA-containing ribonucleoprotein complex export from nucleus | 7 | 2 | 0.17 | 0.01074 |
| GO:0006403 | RNA localization | 9 | 2 | 0.21 | 0.01785 |
| GO:0050657 | nucleic acid transport | 9 | 2 | 0.21 | 0.01785 |
| GO:0050658 | RNA transport | 9 | 2 | 0.21 | 0.01785 |
| GO:0051236 | establishment of RNA localization | 9 | 2 | 0.21 | 0.01785 |
| GO:0042559 | pteridine-containing compound biosynthetic process | 11 | 2 | 0.26 | 0.02644 |
| GO:0042558 | pteridine-containing compound metabolic process | 13 | 2 | 0.31 | 0.03636 |
| GO:0015931 | nucleobase-containing compound transport | 14 | 2 | 0.33 | 0.04178 |
| GO:0000270 | peptidoglycan metabolic process | 1 | 1 | 0.02 | 0.02358 |
| GO:0006027 | glycosaminoglycan catabolic process | 1 | 1 | 0.02 | 0.02358 |
| GO:0009253 | peptidoglycan catabolic process | 1 | 1 | 0.02 | 0.02358 |
| GO:0042133 | neurotransmitter metabolic process | 1 | 1 | 0.02 | 0.02358 |
| GO:0042135 | neurotransmitter catabolic process | 1 | 1 | 0.02 | 0.02358 |
| GO:0046950 | cellular ketone body metabolic process | 1 | 1 | 0.02 | 0.02358 |
| GO:0046952 | ketone body catabolic process | 1 | 1 | 0.02 | 0.02358 |
| GO:1902224 | ketone body metabolic process | 1 | 1 | 0.02 | 0.02358 |
| GO:0006026 | aminoglycan catabolic process | 2 | 1 | 0.05 | 0.04661 |
| GO:0006535 | cysteine biosynthetic process from serine | 2 | 1 | 0.05 | 0.04661 |
| GO:0006584 | catecholamine metabolic process | 2 | 1 | 0.05 | 0.04661 |
| GO:0009712 | catechol-containing compound metabolic process | 2 | 1 | 0.05 | 0.04661 |
| GO:0019344 | cysteine biosynthetic process | 2 | 1 | 0.05 | 0.04661 |
| GO:0051090 | regulation of sequence-specific DNA binding transcription factor activity | 2 | 1 | 0.05 | 0.04661 |
