# Supplementary material for: Arctic charr brain transcriptome strongly affected by summer seasonal growth but only subtly by feed deprivation
Source: BMC Genomics. 2019 Jun 27;20:529. doi: 10.1186/s12864-019-5874-z (PMC6598377; doi:10.1186/s12864-019-5874-z)
Supplement: Supplementary file 8 — Table S6. Biological processes enriched by down-regulated contigs only found FDP versus T0 (see Venn diagram Additional file 1: Figure S1). Terms sorted by the number of contributing contigs. (DOCX 25 kb) [file 12864_2019_5874_MOESM8_ESM.docx]

**Table S6** Biological processes enriched by down-regulated contigs only found FDP versus T_0_ (see Venn diagram Supplementary Figure S1). Terms sorted by the number of contributing contigs.

| **GO.ID** | **Term** | **Annotated** | **Significant** | **Expected** | **p-value** |
| --- | --- | --- | --- | --- | --- |
| GO:0006807 | nitrogen compound metabolic process | 1736 | 66 | 53.76 | 0.03251 |
| GO:1901360 | organic cyclic compound metabolic process | 1400 | 55 | 43.36 | 0.0299 |
| GO:0009058 | biosynthetic process | 1439 | 55 | 44.57 | 0.04759 |
| GO:1901576 | organic substance biosynthetic process | 1374 | 54 | 42.55 | 0.03127 |
| GO:0044249 | cellular biosynthetic process | 1358 | 53 | 42.06 | 0.03699 |
| GO:0006725 | cellular aromatic compound metabolic process | 1375 | 53 | 42.58 | 0.0453 |
| GO:1901564 | organonitrogen compound metabolic process | 620 | 28 | 19.2 | 0.02644 |
| GO:0016043 | cellular component organization | 466 | 23 | 14.43 | 0.01725 |
| GO:0071840 | cellular component organization or biogenesis | 499 | 23 | 15.45 | 0.03484 |
| GO:1901566 | organonitrogen compound biosynthetic process | 451 | 21 | 13.97 | 0.039 |
| GO:0006508 | proteolysis | 298 | 18 | 9.23 | 0.00495 |
| GO:0022607 | cellular component assembly | 222 | 13 | 6.88 | 0.02012 |
| GO:0034622 | cellular macromolecular complex assembly | 127 | 12 | 3.93 | 0.00054 |
| GO:0007017 | microtubule-based process | 132 | 12 | 4.09 | 0.00076 |
| GO:0006461 | protein complex assembly | 173 | 12 | 5.36 | 0.00728 |
| GO:0070271 | protein complex biogenesis | 173 | 12 | 5.36 | 0.00728 |
| GO:0065003 | macromolecular complex assembly | 189 | 12 | 5.85 | 0.01414 |
| GO:0071822 | protein complex subunit organization | 189 | 12 | 5.85 | 0.01414 |
| GO:0006082 | organic acid metabolic process | 226 | 12 | 7 | 0.04764 |
| GO:0019752 | carboxylic acid metabolic process | 226 | 12 | 7 | 0.04764 |
| GO:0043436 | oxoacid metabolic process | 226 | 12 | 7 | 0.04764 |
| GO:0006520 | cellular amino acid metabolic process | 126 | 10 | 3.9 | 0.00554 |
| GO:0051258 | protein polymerization | 36 | 9 | 1.11 | 1.00E-06 |
| GO:0043623 | cellular protein complex assembly | 76 | 9 | 2.35 | 0.00052 |
| GO:0006457 | protein folding | 104 | 9 | 3.22 | 0.00477 |
| GO:0030163 | protein catabolic process | 118 | 8 | 3.65 | 0.02963 |
| GO:1902582 | single-organism intracellular transport | 88 | 7 | 2.73 | 0.01889 |
| GO:0044257 | cellular protein catabolic process | 100 | 7 | 3.1 | 0.03502 |
| GO:0051603 | proteolysis involved in cellular protein catabolic process | 100 | 7 | 3.1 | 0.03502 |
| GO:1901605 | alpha-amino acid metabolic process | 58 | 5 | 1.8 | 0.03302 |
| GO:0006270 | DNA replication initiation | 4 | 4 | 0.12 | 9.00E-07 |
| GO:0006261 | DNA-dependent DNA replication | 5 | 4 | 0.15 | 4.40E-06 |
| GO:0006839 | mitochondrial transport | 16 | 4 | 0.5 | 0.00121 |
| GO:0009069 | serine family amino acid metabolic process | 20 | 4 | 0.62 | 0.00293 |
| GO:0008652 | cellular amino acid biosynthetic process | 31 | 4 | 0.96 | 0.01461 |
| GO:1901607 | alpha-amino acid biosynthetic process | 31 | 4 | 0.96 | 0.01461 |
| GO:0071103 | DNA conformation change | 44 | 4 | 1.36 | 0.04628 |
| GO:0009070 | serine family amino acid biosynthetic process | 7 | 3 | 0.22 | 0.00094 |
| GO:0031032 | actomyosin structure organization | 7 | 3 | 0.22 | 0.00094 |
| GO:0000278 | mitotic cell cycle | 14 | 3 | 0.43 | 0.00828 |
| GO:0006720 | isoprenoid metabolic process | 14 | 3 | 0.43 | 0.00828 |
| GO:0008299 | isoprenoid biosynthetic process | 14 | 3 | 0.43 | 0.00828 |
| GO:1903047 | mitotic cell cycle process | 14 | 3 | 0.43 | 0.00828 |
| GO:0015669 | gas transport | 16 | 3 | 0.5 | 0.01218 |
| GO:0015671 | oxygen transport | 16 | 3 | 0.5 | 0.01218 |
| GO:0006564 | L-serine biosynthetic process | 4 | 2 | 0.12 | 0.0055 |
| GO:0006563 | L-serine metabolic process | 7 | 2 | 0.22 | 0.01809 |
| GO:0006544 | glycine metabolic process | 11 | 2 | 0.34 | 0.04369 |
| GO:0008202 | steroid metabolic process | 11 | 2 | 0.34 | 0.04369 |
| GO:0000281 | mitotic cytokinesis | 1 | 1 | 0.03 | 0.03097 |
| GO:0000917 | barrier septum assembly | 1 | 1 | 0.03 | 0.03097 |
| GO:0006231 | dTMP biosynthetic process | 1 | 1 | 0.03 | 0.03097 |
| GO:0006426 | glycyl-tRNA aminoacylation | 1 | 1 | 0.03 | 0.03097 |
| GO:0006427 | histidyl-tRNA aminoacylation | 1 | 1 | 0.03 | 0.03097 |
| GO:0006545 | glycine biosynthetic process | 1 | 1 | 0.03 | 0.03097 |
| GO:0009157 | deoxyribonucleoside monophosphate biosynthetic process | 1 | 1 | 0.03 | 0.03097 |
| GO:0009162 | deoxyribonucleoside monophosphate metabolic process | 1 | 1 | 0.03 | 0.03097 |
| GO:0009176 | pyrimidine deoxyribonucleoside monophosphate metabolic process | 1 | 1 | 0.03 | 0.03097 |
| GO:0009177 | pyrimidine deoxyribonucleoside monophosphate biosynthetic process | 1 | 1 | 0.03 | 0.03097 |
| GO:0016973 | poly(A)+ mRNA export from nucleus | 1 | 1 | 0.03 | 0.03097 |
| GO:0030186 | melatonin metabolic process | 1 | 1 | 0.03 | 0.03097 |
| GO:0030187 | melatonin biosynthetic process | 1 | 1 | 0.03 | 0.03097 |
| GO:0032506 | cytokinetic process | 1 | 1 | 0.03 | 0.03097 |
| GO:0034754 | cellular hormone metabolic process | 1 | 1 | 0.03 | 0.03097 |
| GO:0042445 | hormone metabolic process | 1 | 1 | 0.03 | 0.03097 |
| GO:0042446 | hormone biosynthetic process | 1 | 1 | 0.03 | 0.03097 |
| GO:0046073 | dTMP metabolic process | 1 | 1 | 0.03 | 0.03097 |
| GO:0048033 | heme o metabolic process | 1 | 1 | 0.03 | 0.03097 |
| GO:0048034 | heme O biosynthetic process | 1 | 1 | 0.03 | 0.03097 |
| GO:0061640 | cytoskeleton-dependent cytokinesis | 1 | 1 | 0.03 | 0.03097 |
| GO:0090529 | cell septum assembly | 1 | 1 | 0.03 | 0.03097 |
| GO:1902410 | mitotic cytokinetic process | 1 | 1 | 0.03 | 0.03097 |
