# Supplementary material for: Arctic charr brain transcriptome strongly affected by summer seasonal growth but only subtly by feed deprivation
Source: BMC Genomics. 2019 Jun 27;20:529. doi: 10.1186/s12864-019-5874-z (PMC6598377; doi:10.1186/s12864-019-5874-z)
Supplement: Supplementary file 9 — Table S7. Biological processes enriched by up-regulated contigs comparing feed deprived versus fed charr at end of experiment. Terms sorted by the number of contributing contigs. (DOCX 22 kb) [file 12864_2019_5874_MOESM9_ESM.docx]

**Table S7** Biological processes enriched by up-regulated contigs comparing feed deprived *versus* fed charr at end of experiment. Terms sorted by the number of contributing contigs.

| **GO.ID** | **Term** | **Annotated** | **Significant** | **Expected** | **p-value** |
| --- | --- | --- | --- | --- | --- |
| GO:0044763 | single-organism cellular process | 2834 | 10 | 6.04 | 0.03531 |
| GO:0055114 | oxidation-reduction process | 404 | 4 | 0.86 | 0.0088 |
| GO:0016310 | phosphorylation | 631 | 4 | 1.34 | 0.03927 |
| GO:0006119 | oxidative phosphorylation | 11 | 2 | 0.02 | 0.00023 |
| GO:0042773 | ATP synthesis coupled electron transport | 11 | 2 | 0.02 | 0.00023 |
| GO:0022904 | respiratory electron transport chain | 12 | 2 | 0.03 | 0.00028 |
| GO:0022900 | electron transport chain | 18 | 2 | 0,04 | 0,00064 |
| GO:0045333 | cellular respiration | 32 | 2 | 0,07 | 0,00203 |
| GO:0015980 | energy derivation by oxidation of organic compounds | 41 | 2 | 0,09 | 0,00331 |
| GO:0010941 | regulation of cell death | 45 | 2 | 0,1 | 0,00398 |
| GO:0042981 | regulation of apoptotic process | 45 | 2 | 0,1 | 0,00398 |
| GO:0043067 | regulation of programmed cell death | 45 | 2 | 0,1 | 0,00398 |
| GO:0006915 | apoptotic process | 57 | 2 | 0,12 | 0,00632 |
| GO:0012501 | programmed cell death | 57 | 2 | 0,12 | 0,00632 |
| GO:0008219 | cell death | 58 | 2 | 0.12 | 0.00654 |
| GO:0016265 | death | 58 | 2 | 0.12 | 0.00654 |
| GO:0046034 | ATP metabolic process | 77 | 2 | 0.16 | 0.01131 |
| GO:0006091 | generation of precursor metabolites and energy | 81 | 2 | 0.17 | 0.01246 |
| GO:0009144 | purine nucleoside triphosphate metabolic process | 82 | 2 | 0.17 | 0.01276 |
| GO:0009199 | ribonucleoside triphosphate metabolic process | 82 | 2 | 0.17 | 0.01276 |
| GO:0009205 | purine ribonucleoside triphosphate metabolic process | 82 | 2 | 0.17 | 0.01276 |
| GO:0009141 | nucleoside triphosphate metabolic process | 85 | 2 | 0.18 | 0.01366 |
| GO:0009126 | purine nucleoside monophosphate metabolic process | 88 | 2 | 0.19 | 0.0146 |
| GO:0009167 | purine ribonucleoside monophosphate metabolic process | 88 | 2 | 0.19 | 0.0146 |
| GO:0009161 | ribonucleoside monophosphate metabolic process | 93 | 2 | 0.2 | 0.01621 |
| GO:0009123 | nucleoside monophosphate metabolic process | 94 | 2 | 0.2 | 0.01655 |
| GO:0042278 | purine nucleoside metabolic process | 99 | 2 | 0.21 | 0.01825 |
| GO:0046128 | purine ribonucleoside metabolic process | 99 | 2 | 0.21 | 0.01825 |
| GO:0009119 | ribonucleoside metabolic process | 105 | 2 | 0.22 | 0.02039 |
| GO:0009150 | purine ribonucleotide metabolic process | 107 | 2 | 0.23 | 0.02113 |
| GO:0009259 | ribonucleotide metabolic process | 110 | 2 | 0.23 | 0.02225 |
| GO:0009116 | nucleoside metabolic process | 112 | 2 | 0.24 | 0.02302 |
| GO:1901657 | glycosyl compound metabolic process | 112 | 2 | 0.24 | 0.02302 |
| GO:0006163 | purine nucleotide metabolic process | 116 | 2 | 0.25 | 0.02458 |
| GO:0019693 | ribose phosphate metabolic process | 122 | 2 | 0.26 | 0.027 |
| GO:0072521 | purine-containing compound metabolic process | 125 | 2 | 0.27 | 0.02824 |
| GO:0006536 | glutamate metabolic process | 1 | 1 | 0 | 0.00213 |
| GO:0006537 | glutamate biosynthetic process | 1 | 1 | 0 | 0.00213 |
| GO:0006562 | proline catabolic process | 1 | 1 | 0 | 0.00213 |
| GO:0009065 | glutamine family amino acid catabolic process | 1 | 1 | 0 | 0.00213 |
| GO:0043650 | dicarboxylic acid biosynthetic process | 1 | 1 | 0 | 0.00213 |
| GO:0006560 | proline metabolic process | 5 | 1 | 0.01 | 0.01061 |
| GO:0009084 | glutamine family amino acid biosynthetic process | 10 | 1 | 0.02 | 0.02112 |
| GO:0043648 | dicarboxylic acid metabolic process | 10 | 1 | 0.02 | 0.02112 |
| GO:0010942 | positive regulation of cell death | 13 | 1 | 0.03 | 0.02737 |
| GO:0043065 | positive regulation of apoptotic process | 13 | 1 | 0.03 | 0.02737 |
| GO:0043068 | positive regulation of programmed cell death | 13 | 1 | 0.03 | 0.02737 |
| GO:0009064 | glutamine family amino acid metabolic process | 15 | 1 | 0.03 | 0.03152 |
| GO:1901606 | alpha-amino acid catabolic process | 15 | 1 | 0.03 | 0.03152 |
| GO:0007050 | cell cycle arrest | 18 | 1 | 0.04 | 0.03772 |
| GO:0009063 | cellular amino acid catabolic process | 18 | 1 | 0.04 | 0.03772 |
| GO:0045786 | negative regulation of cell cycle | 21 | 1 | 0.04 | 0.04387 |
