# Supplementary material for: Arctic charr brain transcriptome strongly affected by summer seasonal growth but only subtly by feed deprivation
Source: BMC Genomics. 2019 Jun 27;20:529. doi: 10.1186/s12864-019-5874-z (PMC6598377; doi:10.1186/s12864-019-5874-z)
Supplement: Supplementary file 10 — Table S8. Biological processes enriched by down-regulated contigs comparing feed deprived versus fed charr at end of experiment. Terms sorted by the number of contributing contigs. (DOCX 21 kb) [file 12864_2019_5874_MOESM10_ESM.docx]

**Table S8** Biological processes enriched by down-regulated contigs comparing feed deprived *versus* fed charr at end of experiment. Terms sorted by the number of contributing contigs.

| **GO.ID** | **Term** | **Annotated** | **Significant** | **Expected** | **p-value** |
| --- | --- | --- | --- | --- | --- |
| GO:0006810 | transport | 1483 | 15 | 8.85 | 0.02027 |
| GO:0051234 | establishment of localization | 1484 | 15 | 8.85 | 0.02039 |
| GO:0051179 | localization | 1501 | 15 | 8.96 | 0.0225 |
| GO:0044765 | single-organism transport | 883 | 14 | 5.27 | 0.00038 |
| GO:1902578 | single-organism localization | 893 | 14 | 5.33 | 0.00043 |
| GO:0015669 | gas transport | 16 | 11 | 0.1 | 3.50E-22 |
| GO:0015671 | oxygen transport | 16 | 11 | 0.1 | 3.50E-22 |
| GO:0006259 | DNA metabolic process | 228 | 6 | 1.36 | 0.00214 |
| GO:0006260 | DNA replication | 113 | 5 | 0.67 | 0.00052 |
| GO:0006270 | DNA replication initiation | 4 | 4 | 0.02 | 1.10E-09 |
| GO:0006261 | DNA-dependent DNA replication | 5 | 4 | 0.03 | 5.50E-09 |
| GO:0051258 | protein polymerization | 36 | 4 | 0.21 | 5.60E-05 |
| GO:0043623 | cellular protein complex assembly | 76 | 4 | 0.45 | 0.00103 |
| GO:0034622 | cellular macromolecular complex assembly | 127 | 4 | 0.76 | 0.00666 |
| GO:0006461 | protein complex assembly | 173 | 4 | 1.03 | 0.01908 |
| GO:0070271 | protein complex biogenesis | 173 | 4 | 1.03 | 0.01908 |
| GO:0065003 | macromolecular complex assembly | 189 | 4 | 1.13 | 0.02546 |
| GO:0071822 | protein complex subunit organization | 189 | 4 | 1.13 | 0.02546 |
| GO:0022607 | cellular component assembly | 222 | 4 | 1.32 | 0.04232 |
| GO:0007017 | microtubule-based process | 132 | 3 | 0.79 | 0.04352 |
| GO:0006231 | dTMP biosynthetic process | 1 | 1 | 0.01 | 0.00597 |
| GO:0006275 | regulation of DNA replication | 1 | 1 | 0.01 | 0.00597 |
| GO:0009157 | deoxyribonucleoside monophosphate biosynthetic process | 1 | 1 | 0.01 | 0.00597 |
| GO:0009162 | deoxyribonucleoside monophosphate metabolic process | 1 | 1 | 0.01 | 0.00597 |
| GO:0009176 | pyrimidine deoxyribonucleoside monophosphate metabolic process | 1 | 1 | 0.01 | 0.00597 |
| GO:0009177 | pyrimidine deoxyribonucleoside monophosphate biosynthetic process | 1 | 1 | 0.01 | 0.00597 |
| GO:0046073 | dTMP metabolic process | 1 | 1 | 0.01 | 0.00597 |
| GO:0051052 | regulation of DNA metabolic process | 1 | 1 | 0.01 | 0.00597 |
| GO:0009221 | pyrimidine deoxyribonucleotide biosynthetic process | 2 | 1 | 0.01 | 0.0119 |
| GO:0009263 | deoxyribonucleotide biosynthetic process | 2 | 1 | 0.01 | 0.0119 |
| GO:0009265 | 2'-deoxyribonucleotide biosynthetic process | 2 | 1 | 0.01 | 0.0119 |
| GO:0046385 | deoxyribose phosphate biosynthetic process | 2 | 1 | 0.01 | 0.0119 |
| GO:0009186 | deoxyribonucleoside diphosphate metabolic process | 3 | 1 | 0.02 | 0.0178 |
| GO:0009129 | pyrimidine nucleoside monophosphate metabolic process | 4 | 1 | 0.02 | 0.02366 |
| GO:0009130 | pyrimidine nucleoside monophosphate biosynthetic process | 4 | 1 | 0.02 | 0.02366 |
| GO:0009219 | pyrimidine deoxyribonucleotide metabolic process | 4 | 1 | 0.02 | 0.02366 |
| GO:0009394 | 2'-deoxyribonucleotide metabolic process | 4 | 1 | 0.02 | 0.02366 |
| GO:0019692 | deoxyribose phosphate metabolic process | 4 | 1 | 0.02 | 0.02366 |
| GO:0006595 | polyamine metabolic process | 5 | 1 | 0.03 | 0.02949 |
| GO:0006596 | polyamine biosynthetic process | 5 | 1 | 0.03 | 0.02949 |
| GO:0009262 | deoxyribonucleotide metabolic process | 5 | 1 | 0.03 | 0.02949 |
| GO:0009309 | amine biosynthetic process | 5 | 1 | 0.03 | 0.02949 |
| GO:0042401 | cellular biogenic amine biosynthetic process | 5 | 1 | 0.03 | 0.02949 |
| GO:0006694 | steroid biosynthetic process | 6 | 1 | 0.04 | 0.03528 |
| GO:0009396 | folic acid-containing compound biosynthetic process | 6 | 1 | 0.04 | 0.03528 |
| GO:0006265 | DNA topological change | 8 | 1 | 0.05 | 0.04677 |
| GO:0006576 | cellular biogenic amine metabolic process | 8 | 1 | 0.05 | 0.04677 |
| GO:0006760 | folic acid-containing compound metabolic process | 8 | 1 | 0.05 | 0.04677 |
| GO:0044106 | cellular amine metabolic process | 8 | 1 | 0.05 | 0.04677 |
